# Supplementary material for: Uvula infections and traditional uvulectomy: Beliefs and practices in Luwero district, central Uganda
Source: PLOS Glob Public Health. 2023 Jun 15;3(6):e0002078. doi: 10.1371/journal.pgph.0002078 (PMC10270344; doi:10.1371/journal.pgph.0002078)
Supplement: S1 Text — (DOCX) [file pgph.0002078.s001.docx]

**S1_Text_Excerpts from Focus Group Discussions with Community Members**

1. BOMBO_FGD_01

…..

Mod: What did you hear about it?

P1: It looks like a pimple. If a child is crying, it elongates and you can easily see it below the throat. I think that is the one we are talking about.

P4: I have heard of it but I am not sure if it is the one. Is it the one where a person experiences difficulty in swallowing food? Is it the one or that is something else?

P8: It is not that one you are talking about. This one is long. It makes a person cough all the time and the child is always crying. That is why the only medication is traditional uvulectomy. It doesn’t cause vomiting.

P5: Is it the one you feel as if dust has entered the throat? Sometimes I cough so much and it itches a lot. It feels like I want to vomit.

P1: It is very common in children, 6 months to I year.

P8: If a parent is reluctant to check her child, it grows and exceeds past one year. The one who doesn’t pay attention to her baby.

P7: The baby always has difficulty in breathing and is always making a clicking sound in the throat (“kukuba mimiro”).

Mod: What about others? Do you hear of vomiting as a sign of akamiro?

P8: No.

P7: No.

Mod: What is this condition called akamiro?

P1: It is a pimple. It is longer than these pimples that attack the skin of person. That is why when they cut it off, it comes with a piece of meat. They use a hooked knife to cut it.

P3: I know of a child who wasn’t talking, they told her she was suffering from akamiro. When she underwent surgery she started talking.

Mod: Where?

P3: In the mouth. The lower one below the tongue is called *akanyata*. For her, it is the long one that they cut.

Mod: Where exactly in the mouth did they cut it?

P3: That long thing in the mouth that peeps when you open your mouth is the one called akamiro.

Mod: What do you and other people of this community understand about akamiro?

P1: It presents with too much cough and the child wants to vomit or make a clicking sound (*kukoona mimiro*) all the time. That is how the mother gets to know that her child is suffering from akamiro.

P2: It is also common among adults who always have cough.

P5: Like I already told you, it just presents in form of a cough and the person keeps coughing endlessly, eventually he/she vomits.

Mod: How long has this condition taken?

P5: It is now 3 months.

Mod: Have you gone to hospital?

P5: No.

Mod: Why?

P5: It was just itching me and I thought I had just developed a cough. So I did not go to hospital.

P2: It is common during cold conditions where the person’s throat is very painful. When it has rains, it becomes severe like allergy. The cough becomes severe.

Mod: P4, what do you understand by akamiro?

P4: I do not know so much about it. Is it the one that swells in the neck or that is something else?

Mod: I came to learn from you, so maybe other participants can respond.

P1: The children with akamiro always undergo surgery at a young age because the parent has investigated and known the cause of the condition. They always cut them when they are young.

Mod: If they haven’t cut it, does it still remains?

P1: No, it doesn’t. It has to be cut out.

P4: Though the parent may try to investigate and he/she finds out that it is not akamiro.

P4: I have never got any chance to investigate this whether the child grows with it or not.

Mod: What about others? What do you think of this?

P8: The child cannot be well until they are 10 years. If you are a responsible parent, you can take the child for surgery. It may even block the child’s throat and they fail to breath and eventually die.

P2: I have my young in law, if he is sleeping he has difficulty in breathing. He always breathes through the mouth. He is 7 years old. Is that called akamiro too?

P8: No that is not akamiro, the problem is with the skull (*ekiwanga*).

P3: I also have that same condition

P1: Kamiro is a very dangerous condition.

P8: I can even lead to the death of a child. The mother may not care what the child is suffering from.

Mod: So is it an ancient disease? I am now over 50 years old and I still hear of it.

P8: Yes it is.

P1: It is the same one. They just hold the child and then cut it.

Mod: Who does it affect the most?

P8: The young children mainly.

P2: The young ones.

Mod: Haven’t you heard it among adults in your community?

P1: I know of a neighbor who had surgery. He was past 10 years old.

P4: My father got sick and they told him that he had *emimiro*. He was given very expensive medicine and he got well.

Mod: What is *emimiro*?

P8: There is a disease called *emimiro* that infects the neck region. Health workers know about it.

P4: That means that was another disease, but not akamiro.

P8: Yes.

Mod: Has akamiro got to do with culture?

P7: It is like other diseases. It does not segregate whom to infect.

P4: I think the same too. It is just a disease.

Mod: Who has heard it elsewhere apart from here in Bombo?

P8: I got married in Tanzania and the kamiro was there too. I gave birth to 8 children while in Tanzania.

Mod: How do they treat it there?

P8: They open the mouth of a child and smear there *akagolo.* After cutting it, they smear there *akagolo*. My child got akamiro, they opened the mouth and used a stick to cut it out. They do not take you to hospital. He became well.

Mod: How is the surgery done?

P8: I do not know but I hear that they just cut it. There is a woman that cuts it. Many people die if it is not cut.

P1: They cut it.

P8: That is the pimple that grows and is cut off.

P5: About my condition, there is a lady who told me to use herbal medicine to smear on the kamiro. I have forgotten its name.

Mod: Did you use it?

P5: No.

Mod: Why?

P5: Because only one person advised me to do so. If many people had advised me to use herbal medicine, the I would have used it.

P6: I have never heard of anyone with it. I just heard that if a child is suffering from it, they experience difficulty in breathing and breast feeding.

Mod: What about the treatment?

P6: They say that they just cut it. There are certain women who know it and cut it.

P4: I heard that when a child has akamiro, they do not get satisfied when they breast feed. The lips of the baby are black. They use herbal medicine to treat it. They keep urinating, and never get satisfied.

Mod: What causes that condition?

P4: You just give birth and find out that your child has this condition.

Mod: What about other participants? What do you think of the treatment for akamiro?

P4: They just cut it (surgery). It is what I hear.

Mod: How do people in this community determine that someone has akamiro and needs attention?

P7: That itchy feeling and excessive coughing makes us think that it is akamiro. And the difficulty in breathing too.

P5: My father has that condition too.

Mod: What about others? What do you think?

P2: Difficulty in breathing most especially the young children.

P1: I heard Hajjat Nakasujja saying that those are pimples in the throat. She might have seen it and talked about it.

Mod: How do you know that the child has akamiro and needs help?

P8: As a parent, you can tell the appearance of the kamiro.

P1: The child is always crying.

Mod: How do you people in this community respond when there is a condition of akamiro in the community?

P1: Things changed, we now take them to hospital

P5: We take them to hospital.

P8: They use herbal medicine, if not possible most people take them to hospital, like Maama Dudu, the Alur woman she treats *binnyo* too.

Mod: Does Maama Dudu just treat it?

P8: She treats and cuts it too. There is another that I hear about.

Mod: What about others?

P7: We tell them to go to hospital.

P4: We tell them to go to hospital.

P7: Me, I think that since it grows in the channel for food and drinks. Therefore the person has to undergo surgery.

1. BOMBO_FGD_02

Mod: As members of this community, have you heard about akamiro in this community?

P7: Yes I have heard about it and seen it before.

P5: Yes I have heard about it. Even my little sibling has ever suffered from it.

P6: Yes, I have heard about it but I have never seen it..

P1: Yes I have heard it but I have never seen anyone suffering from it.

P8: I also heard about it and even I saw where they treat it from both medically and traditionally.

P3: I have never heard about it.

Mod: What is this condition called akamiro?

P7: It is the organ behind the throat, where it starts from. The normal uvula is usually short. Akamiro elongates and grows upwards. That is the infected one.

P2: The one I saw was long and it grows upwards. If the child swallows, it can kill a child. They take it to Mulago for surgery.

P6: I do not know it.

P5: Yes, we grew up knowing akamiro but we did not know what it was exactly. When I read on this consent form I remembered that my little sister suffered from it. It swells and gets pimples on top of it in case you don’t treat it. She told me that if you don’t treat it, it swells.

P8: I have never seen it but I just hear about it. The kamiro swells but I saw a woman who was treated by a traditional surgeon by piercing it.

P1: I heard that a person got infected with akamiro and then she has surgery traditionally. . I didn’t understand how it was but I heard about it.

P4: I heard about it but I have never seen it. Scientifically, it comes from down to upwards. Its long term effects can lead to death and failure to speak properly. I thought that the cause of that is *akanyata*. If it overgrows, there is difficulty in swallowing.

P5: If you are infected with akamiro, there is continuous flow of saliva from the mouth and you cannot swallow.

………………………

Mod: Can you take your children to a traditional surgeon if they were suffering from akamiro?

P1: Like me Nalongo, I cannot take my child to a traditional surgeon. First of all they don’t wash the herbal medicine, they don’t use gloves, and don’t wash hands. But if it is a hospital, I can take him there.

P4: It depends on your income. The traditional surgeon could be cheaper (charges 5,000/=) and yet you can spend 500,000/= when you go to hospitals for surgery.

P1: Remember cheap things are always expensive.

P2: You can leave there your phone as a payment token.

P5: That herbal medicine is ancient. I wouldn’t go to a traditional surgeon. No that you have come, I would first call the numbers on this consent from. Why would I go to a traditional surgeon yet you have taught us about akamiro?

P8: The learned should not take these diseases for granted. T hat has forced people to move from health facilities to traditional surgeons. You should also invent tablets for treatment of akamiro.

P5: The fact is that Akamiro is not a very common disease like malaria and cough. You should trust these researchers now that we have learnt about this disease. They have brought this research to help us.

P4: But they are still researching about it, maybe we should go and teach others in the community about akamiro.

P8: Refer them to hospital. The disease has been around but we do not know how many people have been infected by akamiro.

P4: Please bring nearer health facilities or clinics that can treat akamiro. I am very sure there are very many health workers who do not know about akamiro.

Mod: As a community, how much do you trust the traditional surgeons performing this task?

P8: Since the traditional surgeon is the one who knows it, then you can trust him/her to treat it. Just like the health workers who treat malaria, they are also fully trusted. Someone’s financial status or situation makes the person to trust the traditional surgeon.

P4: You know I am a teacher, it is very difficult for students to trust a new teacher. You the researchers have to work so hard to teach people how to treat it. I personally, currently trust the traditional surgeon because I do not know what solution you have come with to treat akamiro because you are still researching about it.

P8: Just like the traditional birth attendants used to spread HIV among children but after seminars they learnt what to do and began using gloves during child birth.

P4: You are right. Those traditional surgeons should not be eliminated. You should just facilitate them instead because they are already experienced.

Mod: Do you have an idea about how the surgery is performed? Who exactly is involved? What tools are used?

P7: Women are very fearful. We run out and don’t see what happens during surgery. This also happens during circumcision or immunization.

P4: They use a piece of wood to hold the tongue and prevent it from being cut.

P8: There must be some medicine that they put on.

P2: We don’t know exactly what they use.

P4: That remains the traditional surgeons’ experience.

P6: I just hear that they use a piece of wood.

Mod: Are there any costs in this surgery? What kind of costs?

P2: May be 30,000/= or he may ask for 50,000/=. It depends on the way you talk to him or her. It depends on the tone of voice you use when talking to him.

P8: It depends on your bargaining power. They don’t have a specific price.

Mod: Do you think akamiro is associated with TB?

P6: I think so because most people tend to cough excessively without healing.

P2: I think the same too because they keep coughing.

P1: TB and akamiro could be related because every time they cough, the kamiro extends downwards.

P4: I don’t think TB has got anything to do with akamiro considering the signs and symptoms of TB. Besides, the mode of spread of TB is completely different from the one of akamiro. TB infects the lungs. Akamiro could be hereditary.

P8: Akamiro also brings tonsillitis. It could be associated with TB.

Mod: What do you think happens once the akamiro bursts?

P2: It is said that once it bursts, the person dies.

P4: Automatically, once it bursts the person has to die.

P2: The same applies to *ebinnyo*. Once they burst the person dies.

Mod: Is there anything else regarding akamiro that we have not discussed which may be helpful in understanding this condition and dealing with it in this community?

P2: I recommend that you keep teaching us about akamiro. We would like to know its cause.

P4: You should train certain people in differently parts of Uganda on how to prevent and treat akamiro. My question is that what causes this disease? How do they prevent it? Is there a vaccine for akamiro? What is its incubation period? What are the signs and symptoms of Akamiro?

P1: Does akamiro have an association with goiter?

P2: If we find someone who has it, what should we do?

1. BOMBO_FGD_03

Mod: As members of this community, have you heard about akamiro in this community?

P6: Yes we have heard about it.

P1: I have never heard about it

P2: No. I have never heard about it.

P7: I heard that surgery is done in the throat.

P9: I heard about it but I have never seen it with my own eyes.

P7: I have ever seen a child who underwent surgery.

P8: Yes, it grows in the throat.

P5: If a child breast feeds, he/she doesn’t get satisfied.

Mod: What is this condition called akamiro?

P4: I have never heard about it.

Mod: What is this condition called akamiro?

P9: It is said that it is a piece of meat which grows upwards and inwards and stops you from swallowing.

P2: If a child breast feeds, he/she does not get satisfied.

P7: I have heard about it among people from the northern part of Uganda. If surgery is not done, the child does not eat or swallow. I saw a 2 months old child who was suffering from it. They took him to a traditional surgeon who performed surgery on him. They use a razor blade attached to a stick to perform the surgery. The child cannot swallow. It may even chock him/her.

P3: I heard that the child does not get satisfied. They use herbal medicine to treat it.

P6: The only medicine is surgery. That thing is found in the neck. They use a pen like thing with a string, they hold the kamiro and surgery is performed. But I heard that there are organs. One is on the tongue while the other is behind the tongue.

P4: I have never heard about it.

Mod: What do you and other people of this community understand about Akamiro?

P6: My child had *akanyata* below the tongue and she had surgery. The disease is not so common in our community. It is in our region (Busolwe in Butaleja district) where I got married that surgery is performed.

P7: It is common among people from the northern part of Uganda. If you go to Bembe, they are so common there.

P8: I have just heard about it from here.

Mod: Who does it affect the most?

P7: Nubians.

P2: In this community, we have never heard about people who have suffered from it. It is rare in the Buganda region.

P7: I got to know about akamiro because I stayed with the Nubians for some time. It is the father of the child who performs the surgery when a child is born. They have no problem with it. It is their culture.

Mod: Why is the surgery done?

P2: It seems it chocks them.

P7: It chocks the child. When the baby breast feeds, milk is regurgitated. I saw a child of 2 month old who was suffering from it. The child doesn’t have to be sick. It is just like circumcision.

Mod: What do you think? Why do they conduct the surgery among the children?

P6: I think it is a disease. That is why surgery has to be done.

Mod: What would happen if surgery is not done?

P6: If surgery is not done, the baby cannot swallow, eat or drink.

P7: May be it leads to cancer.

P9: As the baby grows, the kamiro grows too.

P2: They prepare the baby so that he/she does not swallow it. If you haven’t have surgery, it can kill you. The moment the baby is born, surgery has to be done.

P8: It seems it is a disease.

Mod: Who does it affect the most?

P7: Surgery is performed when they are still newborns to prevent pain.

Mod: At what age is the surgery performed?

P6: When the baby is months old.

P7: When the baby is weeks.

P9: The baby heals through breast feeding.

P5: I have never seen it. I just hear about it.

P7: That traditional surgery used to be there in ancient years, it has just returned.

P6: You only get to know about these diseases when you get a patient and go to hospital.

1. ZIROBWE_FGD_01

Mod: As members of this community, have you heard about akamiro in this community?

P2: Yes, we have ever heard about that condition. I, myself I have ever undergone traditional uvulectomy.

P8: Yes, we have ever heard about it.

P5: Yes, we have ever heard about it.

Mod: What is this condition called Akamiro?

P2: Akamiro, there is something that they cut in the neck. It looks like a chicken heart. Now, it grows on the throat itself. Every time it grows, it elongates and develops pus. Every time you cough, it itches and if it bursts the person dies. Therefore, when it grows, it falls on the tongue. That is the thing they cut.

P4: When you cough you vomit.

P2: When you eat food you vomit.

P2: Now, that thing leads to a bad feeling in the throat. The child is always suffering from fever, they treat the child but he/she remains sick. But after removing it, it takes weeks or a month. If it is a child or adult whom they have cut, there is always an improvement. You can see that the person is looking healthy. The child becomes pale and anemic. That is how that condition feels.

Mod: But, how does it start?

P1: Thank you teacher. That thing is a created joint. God created it to separated voices, but it gets infected with bacteria. Then they attach to it and start pulling it. The pus that this one (P2) talked about becomes sticky with saliva. It then begins to swell with saliva and touches down onto the tongue. You get the itchy feeling and begin coughing. It also causes swelling in the lungs. Secondly, the longer you take coughing, you develop a disease called “*emmeeme*” in Luganda. This disease causes a lot of other diseases. We have a traditional surgeon who helps us with that condition. He is locates on our village here. He has carried out traditional uvulectomy for so long. Truthfully, he is the only traditional surgeon who helps us with that condition. We have not yet got any help from our health facilities. We haven’t heard any news that a particular health centre carries out uvulectomy. Many people come from far and go to that traditional surgeon because he is the only famous surgeon that is known.

……………..

………

Mod: Aren’t there people who go to seek treatment directly from the traditional surgeon without going to the hospital?

P1: It is not easy.

P3. No, it is not easy. Most of them first seek for treatment from the hospital before they go to the traditional surgeon.

P1: I also think the same about it. Once the person is suffering from cough, the first treatment is to go to hospital. If treatment fails, we start investigating why the cough isn’t disappearing. That is when the advisors tell you to take the person to the traditional surgeon. That is how we discover that condition.

Mod: What do others say about it. You may be having a different explanation. Please tell us everything. Don’t keep silent. Isn’t there another method apart from surgery?

P5: We haven’t discovered another musawo who can treat it differently. He is the only one we have right now who can remove it incase all treatment in the hospital fails.

Mod: P2 as you were away, we asked why should the patient suffering from Akamiro undergo surgery?

P2: Akamiro is like hernia where we haven’t yet discovered the true medication/treatment. Removing is the only way to treat it currently. May be the research that you have conducted about akamiro will help us and the government should do something about it so that it can be treated. Truthfully, akamiro is not only here in Buganda, it is also there in West Nile. My in-law, the traditional surgeon learnt how to conduct surgery from West Nile, it is where he was born. It means it doesn’t have medicine, its only treatment is to remove it surgically. That is how it is.

Mod: Thank you so much. Do you think carrying out traditional uvulectomy has got anything to do with culture?

P2: No, it has got nothing to do with culture because we do not know who invented this traditional uvulectomy. In most cases, traditional surgeons copy from each other for example in this area of CCCCC and XXXXXo, that traditional surgeon trained his son. That means if he dies, his son will be the one to do the traditional uvulectomy.

Mod: Does that mean his son also does traditional uvulectomy?

P2: Yes, he is the one who trained him. That means it is not to do with culture. Even in Bugerere where my wife comes from, there used to be a traditional surgeon but he later died. So that is how it is. It has got nothing to do with culture.

Mod: What about others?

P5: The way he has explained it is how it is.

Mod: Any additions?

P3: It is not easy to treat. Not everyone one can do traditional uvulectomy. That explains how complicated this disease is. In this area, we have got only one traditional surgeon who does this work. People come from all over. If there was as traditional surgeon in Kikyusa and Kyaggwe, those people there wouldn’t be coming this side. It doesn’t need witch craft to treat it. The job is not easy because it involves cutting something in the mouth, which is very risky. It involves accidents, you may make a mistake and the razor blade is swallowed or it cuts the cheek by mistake. That is why the traditional surgeons who treat it are very few.

………

Mod: As a community, how much do you trust the traditional surgeon performing this task?

P8: Yes we trust him because he does not ask you to buy any medicine from hospital. He tells you to chew hard corns and insert cotton wool in the ears and then you get well, unless you have got another disease.

P3: Thank you musawo. We trust him 100% because he has been treating us for very many years. Secondly, none of his patients have died after surgery. Therefore we are not fearful, because all the people that go there are treated well and heal. So we trust him 100%.

P2: Since he is highly experienced, after surgery the patient is fine. There is a 2 month old baby they brought to him and he operated on her. That means he is highly experienced. Just like this gentleman has said (P3) that no patient has died after surgery. I have never heard any problem arising ever since he started conducting surgeries. After surgery, within 3 days the person is okay and has healed. The cotton wool he tells you to insert is for preventing air from entering the ears. After 3 days, the person is fine, so we trust him.

P7: I am asking, can’t he train me to do traditional uvulectomy?

P2: It depends on how much he will charge you. You can go there and consult him.

Mod: What is the use of the cotton wool after surgery?

P2: It prevents cold air from going into the wound after surgery.

Mod: What happens if that air enters into the ears?

P3: Once, the cold air enters and blows over the fresh wound, it pains a lot. You can close the mouth to prevent air from entering, yet you can’t close the ears. That’s why he inserts a piece of cotton wool.

P5: In addition, closing the ears with cotton wool prevents continuous coughing . If you do not chew hard corns, he tells you to drink maize porridge for 3 days.

Mod: What about others? What do you say about trusting the traditional surgeon?

P8: We trust him a lot.

Mod: Why?

P8: Because he treated my children very well.

Mod: How much did he charge you?

P8: Around 5,000/= , 10,000/=. He has increased with time. He now charges 30,000/= like that.

P5: In addition, we trust him because he has treated very many people and none of them has died. People go to hospitals for surgery, patients die after but with him, nobody has died.

Mod: Please explain how the surgery is performed?

P2: He breaks the new razor blade into small pieces and remains with the corner of the razor blade. He then breaks the corner of the stick, inserts the razor blade and ties it with cotton. Then he gets a pen stick attaches it to a wire and he tells you to open your mouth if you are an adult. After opening the mouth, h inserts the stick carrying the wire into the mouth, pulls the wire, he then inserts the stick having the wire into the mouth and cuts of the uvula. The uvula is not so big, after cutting it off it comes along with the wire.

Mod: Does he cut it all completely?

P1: He cuts the uvula and shows it to you after.

P2: It takes approximately 2 minutes.

Mod: What do you think of that kind of surgery?’

P.3: It doesn’t have any effect among the people. Good enough there is no extra medication required apart from the hard corns cotton wool. There is no extra labor needed such as treating the wound. He removes it and you pay him after.

1. ZIROBWE_FGD_02

Mod: As members of this community, have you heard about akamiro in this community?

P6: We have been suffering from the disease, however we have been using traditional surgeons and not trained health care workers. We travel long distances to go where these traditional surgeons are located, to take the patients suffering from akamiro. We have been trying to go to CCC , FFF and GGG . They have been treating these patients very well. This proves that they are experts because the patient heals properly and the disease completely disappears. That is all I know about akamiro. One of my sons suffered from it, I took him to a traditional surgeon in xxxbx. He is called ddddd. He is an alur, he was the first traditional surgeon to treat for us that disease.

P7: I know that disease very well. If the person is suffering from it, they cough and vomit excessively, it keeps itching them in the throat. I am the one who checks for it in this community (*mukebezi)*. When a person comes to me complaining about akamiro, I check it to see if it is the one or not. If it the one, I refer the person to the traditional surgeon. After surgery, I inquire about how the person is feeling. He tells me the person is in good condition and healed completely.

P5: My child has ever suffered from it. I went to the hospital to seek treatment, but the problem is that the trained health workers have no knowledge about it. They present with fever and excessive cough, until you get a person who advises you to go to a *mukebezi* to check if it is there. Just as the lay has said (P7), when she checks and it is actually there, she refers you to a traditional surgeon. In vvvvv, he is called Mr. xxxx. He is famous for traditional uvulectomy. After surgery, the fever reduces though the child cannot breast feed well immediately after surgery.

P4: Me too, I have a child who was suffering from it. I took him to a traditional surgeon, surgery was done and my child is now fine. He is now 25 years old. The traditional surgeon who treated him was a woman who was staying in cccccc that time. Kccccc is located here in Luweero. But this female traditional surgeon passed away.

Mod: Did she leave a successor?

P4: No.

P7: Every traditional surgeon has their skills on how they conduct the surgery.

……..

Mod: Is it common in certain cultures?

P6: No, it affects all cultures.

P9: Just like COVID affects people from all cultures.

Mod: How do people in this community determine that someone has akamiro that needs attention?

P9: As long as the person has been checked. The good thing is that we have got a traditional surgeon in the area. We pray that God continues to keep him alive.

P3: It presents with signs and symptoms.

P6: Such as coughing and itching in the throat.

P1: And severe fever among the children.

P7: There is profuse vomiting among children. They keep regurgitating the breast milk.

Mod: How do you and people in this community respond when there is a condition of akamiro in the household/community?

P7: I check them (*omukebezi*) and recommend them to the traditional surgeon for surgery.

P2: I direct them to the traditional surgeon for treatment.

P6: I send them to the traditional surgeon who confirms that it is really there and it is cut out.

P9: I also refer that person to the traditional surgeon for treatment in order to save the child or adult’s life.

P8: But the traditional surgeon is really expensive. He charges between 60,000/= to 70,000/=.

P5: He used to ask for a hen and money. Now he no longer ask for a hen.

P1: We are not even sure whether the razor blade he uses is new or used.

Mod: Doesn’t he unseal it when you are there?

P6: He breaks the razor blade in your presence. Then he attaches a razor blade to a string.

P7: He uses a string and razor blade and within minutes the kamiro is cut out. They are highly experienced surgeons. When it comes to the cost, the children are charged 30,0000/= to 50,000/=, adults are charged 70,000/=. It depends on the financial situation in are in that time.

Mod: Do you have an idea why surgery has to be done?

P2: Because it is the only mode of medication used. Traditional surgery is the only method we know that can relieve the pain of akamiro. It is not that we can use local herbs such as mululuuza.

P5: It is because the trained health workers in hospitals do not know much about it.

P4: Traditional surgery is the only medicine. When you get well you feel better.

P9: It is the only available medicine to save life, because there are no tablets available to treat it.
