# Supplementary material for: Uvula infections and traditional uvulectomy: Beliefs and practices in Luwero district, central Uganda
Source: PLOS Glob Public Health. 2023 Jun 15;3(6):e0002078. doi: 10.1371/journal.pgph.0002078 (PMC10270344; doi:10.1371/journal.pgph.0002078)
Supplement: S2 Text — (DOCX) [file pgph.0002078.s002.docx]

**S1_Text. Excerpts from Key Informant Interviews with Community Health Workers**

1. BOMBO_KII_01

………….

I… Out of 100 people how many do you think have it?

R: For me i think it’s like 50 percent…..

I: …. like 50 percent?

R: Yes

I: among the adults?

R: Yes

I: eeeh okay

R: Because when you are with them, they say for me they cut me, they told me they are going to cut me because there is even a man that they cut in October but by the time they cut him he had diabetes, so he died in the process. They cut him from the GMH, no they first cut him from the local surgeons, and he failed to recover so they took him to the GMH and spent there like 2 weeks then died.

I: So, were they going to cut him from the GHM?

R: No for them they do not cut, even the other lady I told you about was referred to Mengo for them they treat the disease you have but do not cut it.

I: You talked about the different medications, you talked about the local surgeons, the modern doctors, and the traditional herbs they take. How do the traditional herbs work?

R: For me I have my son, he at times struggles with it with a funny sound, it is disturbing him, but they bring for him herbal medicine, but I do not see any change but when he has grown time comes and he coughs then saliva chokes him because he is now in senior 3, saliva chokes him. I think this is caused by that disease of the uvula because when he sleeps at night, you then hear him waking up suddenly and coughs as if saliva has choked him. Then I ask him what is wrong, he says saliva is choking him.

I: Have you ever checked him to see if he has Kamiro disease?

R: Personally, for me i have never checked him but when you started your research, because I have ever taken him to xxxx regional referral hospt in the ENT Clinic, then the Doctor told him that his thing is weak….

I: …The uvula…

R: …yes, it over shakes, he then gave me medicine and he seemed like he was healing but right now when he has grown up, he is 15years going to make 16 years but now it is over disturbing him, by the time you wake at night you hear him coughing too much and you ask him what is wrong, he says the saliva has choked me.

I: Okay, so how long has he been like that, that boy?

R: From day 1….

I: …. From birth?

R: Yes

I: How old is he now?

R: He is going to make 16 years.

I: But doesn’t it disturb him like when he goes to sleep, does he get sleep or does he snore?

R: He snores and even when he was young you might think there is a grown-up person, At times he gets defeated and makes a funny sound. There is even another child who has it, so I found when that woman’s child also does the same thing as my son but I don’t know whether they checked him from referral hospt and I also didn’t take time to check him but when you started doing this research, the very first time you came, that when I said, this child might be having that disease then I went to see her mother to check whether he has it, but I didn’t find her there.

I: Okay. So, depending on your skills, Among the households that have the Kamiro diseases, for them what do they tell you as the VHT looking after them? These homes you have gone to that have kids with Kamiro disease, what do their parents tell you as their immediate nurse.

R: Now for them what they know is that modern Doctors can not treat that thing, for them they think its treated by traditionists and the ones that cut it, that is the only hope they have.

……

I: Okay, So, you have said that when they come, you refer them to the local surgeons not these modern Hospitals, so when you tell someone to go to the local surgeon, how does he passive it?

R: They also passive it like that that modern health workers do not have medication which…, because even when one comes here [health centre], he will make a line and they will end up referring him there [another facility], even there, they will write for him a letter referring him to Mulago [National referral hospital]. So, when he sees that process of being disturbed, he just cuts it short and goes there [traditional surgeon].

I: …so when you refer them to the local surgeons, that is where they go.

R: Yes

I: So how do those local surgeons deal with it? when they go there, do they get the correct treatment they are supposed to get or?

R: The people they treat, get healed. For example*, my brother, he was cut and now he is healed*. My husband was cut but for him he did not heal. When they cut him, I used to ask him whether the wound had healed… i first sent him to the GMH and when he went there, they prescribed for him some medication which he took but still he did not get any improvement. Then he started getting swollen here, in May last year then I told him to go and see the major ENT who works on my kids, he is a doctor, so he went there, and he prescribed for him some medication so he went back after completing it, he went back but he told him to go to Mulago so that he can be checked again. It seems when he went to Mulago the process was long, so he went to xxxxx hospital and they did all the tests again, by then he was not on any medication and the whole of here was swollen…

I: … the throat was swollen? …

R: … Yes so, they performed a CCT scan on him and checked everything, all the general oral tests and when they finished, they told him that he has cancer….

I: …. hmm..

R: But it is at its first stage, they then told him that he will be fine and checked his organs and all were okay. They told him that he does not have any problem, but this went on and worsened, he even failed to eat, they even made a hole here and was being fed from here...

I: … on the stomach….

R …yes, but it persisted, and his condition worsened….

I…when the throat is paining him…

R: … Yes, he could not even talk, he would talk but was just struggling with breath, it seems he no longer had any space for breathing, then they took him back, when they were going to make a hole hear, when they gave him cauliform, he did not regain his cautiousness….

………….

1. BOMBO_KII_03

……………….

I: Okay, now which medicine do these local surgeons give to calm down people’s pain? Or there is no medicine they give?

R: Ehhhh, when they finish cutting you, for us we call it combo, we put rock salt, Ash, we put every thing and the container starts to leak, that is what they…..

I: …. this water that is dripping…

R: …yes that is what you take, in 2 days you are okay.

I: Are there people who get septic wounds?

R: No, I have never heard of it.

I: Hmmm…

R: And that is the treatment, you just take combo, and you heal, I think it is because of the salt content, its God who knows that.

I: So, combo contains ash and rock salt?

R: No, only ash, they make it in a way that it keeps dripping, the water goes through the ash and it drips out, if it is a child, they use a spoon, a grown up just drinks, for 2 days and you are okay…

I: …2 days when you are healed…

R: ..yes in 2 days you are okay.

I: These people whose uvula has been cut, does it heal completely, or they continue getting complications?

R: No, they do not, it heals completely, it does not grow again. Before cutting it, coughing is too much and it does not stop, they do not eat, drink but afterward, he starts eating and drinking.

I: You said when people come to consult you when they have the disease of the kamiro, you send them where they cut it from, how do they perceive it when you tell them to go to someone to cut it?

R: They go there, and they cut them, they do not have any problem with it, and when they go there, they cut them and they heal, there has never been death.

I: How do you get that strength, to tell those people that they should go, and they cut them?

R: It is because ever since I have lived, that person is cutting and there has not been any case of death and yet he is cutting, so that is how I get the strength to send them there.

I: Hmmm, so the fact that ever since you were borne, they have been cutting and there are no death cases, so this gives you the strength to refer people there to be cut?

R: Yes.

I: So how do the different house holds treat their people who have the disease of the uvula, how do they treat them, do they separate them from others, does he get special treatment how do they do it?

R: They treat him properly because they know that that is the disease of the uvula and they rush, he cannot spend 4 days when the cough is too much, even when you are grown up. Now like that youth, by the way its his colleague who told him, it was not even me. He told him that lets go try and see and when they reached, they checked and told him that it is the one.

I: Okay, so when they realize there is a child who has Kamiro disease, who do they consult first? Do they first consult from you the VHT, some one in the home, who do they consult first…?

R: To confirm that it is the one?...

I: ..yes..

R: Hehehe, the parents know it, the moment you cough for some days, they tell you, open your mouth and we see, because you can see it, they just use a torch, and they see that it is the one.

I: ..so do all the parents know it?

R: Yes, they know it

I: You mean there is no one who does not know it?

R: They know it, once you cough too much, they start saying it might be this, then they tell you open and we see, they then confirm that it is the one. There is no parent that does not know it…

I: Okay…

R: ..the main thing is when you cough without stopping, your friends are sleeping and for you you are coughing, no, the first thing they say is, hope it’s not kamiro disease…

I: Okay...

R: …if its not the one, that when they go to hospital.

I: Okay. For the parents that have kids with kamiro disease, what do they fear most about this disease of the uvula? What makes them fear it most?

R: Its death, because it kills, for example if it’s a child, he doesn’t eat, drink, if he breast feeds, he fails to breast feed but just coughs, he cant even take 4 days. They can be patient with the first day, then the second day, since the parent also cannot sleep, that is what keeps in her head, asking herself questions….

I: ..meaning when this child catches the kamiro disease, he can’t sleep?

R: He does not sleep and even the mother does not, he just keeps coughing without stopping, he coughs too much.

I: Hmmm, so as we are concluding, in your own thought as VHT Jxxxx, how is the disease of the uvula supposed to be treated in the household.?

R; In the household? the Kamiro disease? these people should be given proper equipment to use….

I: …which people? The local surgeons?

R: Yes, those ones who cut it in villages, should use medication…

I:.. the modern medicine or traditional…..

R: Even if its modern but also this other equipment they use, they should be stored well. Because one can be with his knife for years without changing it, that is why the government should help them, even with gloves because they do not use them….

I: ..so they don’t even have gloves?

R: ..No…

I: So when you come what do they do, do they wash their hands and they work on you? What do they do or they don’t even wash their hands….

R: .. before Covid, they did not even wash their hands…

I: ..you just came opened your mouth and they cut you?

R: Yes, let me see, and how many people does this piece of wood, work on? Because I do not see any other thing that they do, apart from that thing that cuts and the wood and putting that ash in the wound.

I: Hmm, okay. By the way, when some one goes to those local surgeons, what does he pay, or its for free?...

R: ..for free, can a doctor work for free, its 20000 shillings…

I: …so every patient pays 20000 shillings…

R: …yes..

I: ..when you don’t have it they don’t work on you?

R: No, the other one is a job, 20000 shillings.

1. ZIROBWE_KII_01

………..

I: From your experience in dealing with households that have cases of uvula, what do they tell you as VHT?

R: In the first place if a child or old person contracts uvula, he gets itching then coughs and then starts vomiting, so before the surgery is performed he cannot stop coughing, but when the uvula is cut he stops the coughing and vomiting.

I: Does this coughing take place at night or during day?

R: The coughing is consistent for both day and night.

I: How is the appetite of the patient during this time?

R: He eats with pain not like a normal person.

I: What do you mean like a normal person?

R: Am trying to say that he does not have test for food, he is just eating not to die of hunger.

I: You mean he is not enjoying?

R: You see whenever he eats, he vomits the food.

I: As a VHT, how do you help them when they bring forward this condition of uvula?

R: Currently such people have not been coming to us.

I: Whom do they go to?

R: They directly to the traditional surgeon, then after examining the patient and confirming that is when he performs the surgery.

I: In case they come to you what would you tell them?

R: I would just direct them to go for uvulectomy since there is no any other treatment for it.

I: Do you know the surgeon, so what would you tell them?

R: Yes, I know the surgeon, so if a patient came to me I would direct him to the surgeon.

I: How long is it from here to the surgeons place?

R: It is less than a mile.

I: When these people come and you send them to the surgeon, do they go there?

R: Yes, they do go there then after some time you hear that so and so is now fine.

I: Are there some who go and get partial uvulectomy?

R: No, there is a way he ties the uvula then he cuts it and pulls it out using a string.

I: Are there some people who die after the uvulectomy?

R: Nobody has so far died from there.

I: Do all the people you recommend to go to the surgeon go there?

R: They all go there.

I: Do they have confidence in this surgeon?

R: Due to the pain a client is going through, and that the parent has nowhere to take the child, all she has to do is to trust the surgeon.

I: In your view, how should this condition of uvula be dealt with by the households/ individuals?

R: The bad thing is that we have never been taught how the uvula infection comes up, we need to be taught more signs and symptoms of uvula such that we are able to identify it in people and send them to hospitals for treatment at an early stage.

I: Why is it that when they come to you now you do not send them to health facilities instead of sending them to surgeons?

R: They first go to health facilities and are given medication for cough and they do not heal and remain coughing and vomiting but when they go to the traditional surgeon for examination he cuts it and heals permanently.

I: Are trying to say that the health workers cannot carry out the surgery? Why are sending them to traditional surgeons?

R: I have never heard of anyone who was operated upon by health workers in the health facilities.

I: When you send them to the health facilities, does the medication not work on them or?

R: The medication served in health facilities does not heal it.

I: What kind of medicine is usually given to them?

R: They at times give them tablets or syrup but still they remain vomiting and coughing and feeling the itching on the kneck.

I: How does the young child feel when he is suffering from uvula?

R: He might fail to breast feed at times and forced to take milk and when he coughs he vomits.

I: When those children vomit the milk, do they lose weight?

R: They lose a lot of weight

1. ZIROBWE_KII_02

………………….

I: In your service as a VHT in this community interacting with community members, how often have you found households whose children have uvula?

R: It is rare to get people suffering from uvula, they would bring children complaining that they are suffering from cough and give them the cough medication which is amoxicillin, then for uvula they get other people who advise them to go to different people for treatment. I personally only give them medication for cough. What people do here is that they recommend to go for examination of uvula at mukyala nxxxxxxx and when it is confirmed then you are sent to the local surgeon for uvulectomy.

I: You talked about mukyala nxxxxxxx, what is her role?

R: She is a normal lady on the village though she is specialized in examining and identifying the uvula. Then there is a surgeon called xxxxxxe in Kccccccccc who does the surgery.

I: What is the category of people who usually suffer from uvula?

R: Any one can suffer from the uvula, there is no specific age, it affects both the old and the young, like I had a neighbor who a prolonged cough and people had said that he was suffering from TB, then he was taken for examination and it was confirmed and later underwent surgery and he healed, he never coughed again, he just died recently of a different infection.

I: You said that man coughed as somebody who is suffering from TB, How is it like?

R: It is severe prolonged cough, for the child he may even vomit the breast milk after taking it, the uvula as the child coughs it knocks on the throat and end up vomiting. \

I: So if a child breast feeds and vomits, does it mean he/she is suffering from uvula?

R: No, it is not that, it might be a strong consistent cough which is prolonged for like two weeks, then the child is taken for examination from a specialist and when it is discovered the child is taken to the traditional surgeon for surgery. Most people go to Kxxxxxx in fffffff for the operation, there is also another surgeon in Wxxxxxx x village called yyyy who also operates the uvula. But basing on how they cough it is what they base on to take the child to the person for examination after failing to heal under medication and when it is discovered the child is taken for operation.

I: From your experience in dealing with households that have cases of uvula, what do they tell you as VHT?

R: It is the chairman who had children suffering from uvula, so he has more experience in dealing with it, he is actually one of the examiners of uvula in our area. So for my case am not much experienced in it. Our chairman has had over 5 children of his undergo uvulectomy.

I: As a VHT, how do you help them when they bring forward this condition of uvula?

R: They do not bring the child ad say it is uvula, we usually handle children under 5 years, they bring the child when is coughing, so I give the child medication for cough, I usually as for the age of the child and for how long the child has coughed then I give the medication for cough which is amoxicillin. It is after failing to heal that they decide to take the child to the surgeon, so to me they bring somebody suffering from cough not uvula and I give medication.

I: So when you give them medication and it fails, what advice do you give them?

R: I refer them to a higher level facility not to traditional surgeon, if I do not refer to a HCII I then refer to HCIII. If they decide to go to clinic it is ok with me but I request they bring back the referral form which is at the bottom of the form.

I: How do they respond to the advice of the referral?

R: They usually take at in good faith and go to the health facility, so from the facility I do not know what happens next, all I want is my referral form returned.

…………………………
