# Supplementary material for: Uvula infections and traditional uvulectomy: Beliefs and practices in Luwero district, central Uganda
Source: PLOS Glob Public Health. 2023 Jun 15;3(6):e0002078. doi: 10.1371/journal.pgph.0002078 (PMC10270344; doi:10.1371/journal.pgph.0002078)
Supplement: S3 Text — (DOCX) [file pgph.0002078.s003.docx]

**S3_Text_Excerpts from Transcripts of IDIs with Uvulectomy clients and caregivers**

1. BOMBO_IDI_Client 04

…………….

I: ..so do they give you a syrup which is sweet, a traditional syrup, which one do they give you?

R: They give me tablets...

I: …did they tell you the name of those tablets?

R: ….i think, its vitamin c…

I: …eeeh, so they gave you vitamin C?

R: yes, but there were others…

I: …those others that they gave you, were they capsules or, were they the capsules that have red and black or?
R: …..yes, those exact ones.

I: So they gave you vitamin c and capsules….

R: … yes...

I: … did they tell you they were going to help you with the cough?

R: ..yes, but I swallowed and didn’t realise any change.

I: So, for how long did you swallow those tablets, and you did not have any change?

R: I took the whole dose and completed it….

I: So how long did it take you to complete the dose, is it a week, month or?

R: It is a week.

I: So, you used only one dose?

R: yes.

I: So, when it failed to work on you, didn’t you try to go to the hospital so that they can check you, either for TB, or anything?

R: No, I did not go.

I: So now, you see yourself in the mirror and realise that its black, what followed?

R: I then said that I had to go to those doctors that cut….

I: …hmm..

R:… I appointed him a day and then he came and cut it.

I: So now, you made an appointment with the doctor, what time did you go there for the doctor to cut it?

R: I informed him in the morning, and he told me that they usually cut this thing during morning hours or in the evening when the moon is down.

I: Why do they cut it in the morning and evening?

R: I do not understand why, but that what he told us.

I: So when you go there in the morning?

R: They cut me in the evening, because when I went there in the morning and yet I was still at work then I decided that at least in the evening….

I: ..in the morning you were still working, so you decided evening after work…

R:.. yes...

I: What time of the evening did you go there?

R: It was around 5 pm…

I: ..so when you reached, did he work on you immediately or you first waited?

R: He worked on me immediately.

I: What equipment did he use when he was cutting that uvula?

R: Equipment, one is like wood and the other is a metal with a hook that he uses.

I: So what does the wood do?

R: It catches the other thing so that it does not fall in the throat….

I: ..so he puts the wood in the mouth to obstruct the throat so that when he cuts with the hook, he just pulls it out, as the wood is obstructing the throat so that it doesn’t fall there.

R: This thing is like poison, if it falls into the throat, you can even die, that is what they say…

I: …so if he cuts it and it falls inside and you swallow it, you might die…

R: …yes.

I: ..hmmm, okay.

R: So that wood helps them to catch it so that it does not fall inside…

I: ..hmmmm, so he used a hook and a wood. Is that hook like an iron sheet, wire, how is it?

R: Its long like let me say a spoon…

I: …flat like a spoon…

R: …its long, as you see a straw ….

I: Is it that hook that cuts?

R: Yes, it is the one that cuts, because its curved….

I: …so when he puts it inside, he cuts and removes it…

R: …yes..

I: ..ehhhh, so when he is using this hook to cut, does he use it on every one, or?

R: It seems it is the one they use, but there is away they handle it….

I: …..hmmmm, do they wash it?

R: Yes

I: What does he wear, does he wear gloves or nothing?

R: He puts on gloves…

I:….when he is going to work on you….

R: ..yes.

I: Hmmmm, does he give anaesthesia you?

R: No…

I: ..so don’t you fear when doesn’t paralyse you?

R: No, he just cuts you, like that...

I: …do not you fear, for example for you they have just cut you, don’t you fear?

R: Now like me, I can be wanting that thing to be removed...

I: …. because you are already fed up with it…

R: …so I give up on anaesthesia….

I: ..hmmm, so he has finished cutting the uvula, what did he give you to ensure that blood doesn’t come out too much?

R: He gave me lemon…..

I: ..Lemon?

R: ..yes

I: What did he tell you to do, did he remove the juice and gave it to you, how was it?

R: He removed the juice and squeezed it properly into the cup, then removed the seeds and I then drunk it.

I: How many spoons were they?

R: It was one lemon…..

I: ….so you took juice that comes from one lemon.

R: Yes...

1. ZIROBWE IDI_Client 01

……….

I: So Mr. xxxxx, tell us about what you went through, how was it when you suffered from Kamiro.

R: Me to know that I was suffering from Kamiro, it started when I could not swallow the saliva well and I also got cough, coughing every time, then my wife told me may be you got Kamiro but I tried telling her that no our clan doesn’t suffer from Kamiro that’s for you people from other tribes, she told me good enough the person who treats those uvulas is our own let’s try and see that they examine you, but I resisted for like three more months not accepting my uvula to be examined, but she insisted on me for some time after then I accepted and went there and I was examined, after I was told it’s there, then I went back and narrated it to my father and he told me no, us the Baganda we don’t suffer from Kamiro its always common among those other tribes, then I told him I have been examined and it’s there, it took like six months but whenever I would swallow saliva it could not go and would also cough endlessly. It reached a time and I surrendered on my own, I called my wife and told her tomorrow morning am going to Muzeyi to cut that thing out of me. Then she told me that I have to take with a new razorblade when coming, you buy it yourself you we don’t want to find it at his place. So I got up early morning I rode my motorcycle up to Muzeyi’s place, he asked me for fifty thousand shillings but he again said since am his son I will treat you with whatever you have in your pockets. He then asked have you brought the razorblade, and I told him I have it Muzeeyi, and he told I want you to unwrap it yourself then I unwrapped that razorblade very well and handed it to him. He had told I have to very early morning before eating anything and still I went very early by the time it clocked 6am I was already at his place, he gave a chair to sit and he also prepared himself he washed his face and came, he then gave me a mat and told me to lie facing upwards, then I very well lied facing up. He first came and told me to widely open my mouth , and I widely opened my mouth and he told me but my son the thing was going to burst and cause you great problems. So after he was done there, me lying facing up to then told me have you gained the strength my son, he asked that three time and I replied him am strong, then he told widely open your mouth and I widely opened mouth, by the way its serious opening of the mouth where the jar stretches very well. But I did not see the away he tied the razorblade...

I: ...the razorblade?

R: ...yes, because my heart started fearing was feeling some fear in my heart, then he put like this, he put just deep in my mouth and he hooked it and removed and he dropped down there, but that thing was very big it was the size of his finger of mine...

I: ...this middle finger...

R: ...yes the middle one ...

I: ...the width...

R: ...yes the width...

I: ...the length?

R: No, but the with is like of my middle finger, but he told me it was going to burst and said in case it bursts sometime it kill a person. There is some medicine he gave me but I finally realized it salt.

I: Our normal salt put in food or the Kisula?

R: It was kisula, I realized it because he tried to hide it from me but my brains found out that it is a Kisula. He then gave it to me and told me I should be sucking it and swallow, and then I swallowed. He then asked did any one drop you here I replied no, I rode myself on the motorbike. He told me get a cloth since the time is still morning we don’t want the air to blow through your ears and so I got my jacket and put it in a way to be able to protect my ears from the air.

I: The Jacket you are talking about, you tied it round your ears?

R: Yes I tied around my ears because he told me it’s not good for the air to blow through you. He told me when you reach home get this salt which we eat and put it in warm slightly water such that you be swallowing that water little by little, so when I reached home it’s what I did. But that thing took me like four good days, they were four days and my eating was not much when I eat I could feel the food scratching me where they cut. I even got some fear and I called him and told him Muzeeyi but me I always hear people telling that the those you work up on only spend just two day then start eating but for I feel I can’t eat, he told me my son be strong I have experience in what I did you are not the first and I believe you’re going to be alright. And also since my wife was confident on what was done to me, she told me be strong you are going to get well. So I bought for my people meat for Easter, for them they ate very well but would try telling me please try to eat but Muzeeyi had told me try to take that soup when its warm since it always has some salt in it, and for it I used to try it with a spoon but sincerely speaking I got some fear, I used to get that spoon and keep taking that spoon. After like four days I would even eat a piece of matooke, I was well. So since then I have never that thing in my throat even up to now am fine.

I: You have mentioned about the Kisula((rock salt)) salt,

R: Yes

I: What was the purpose of the Kisula((rock salt))

?

R: I don’t have facts about it...

I: ...had he told you that it will help to reduce on the bleeding?

R: Yes, but the blood came because immediately he finished he told to spit three times, he asked me is the blood coming I replied no it is not coming, i spat for the first time but the blood was not all that much. Of course he gave me what he called his medicine but me on looking I realized it was kisula salt, and he told me keep sucking that salt little by little but he had grinded it very well not like how the kisula always look. That’s how I was.

I: Okay, now you mentioned about your wife and also you mentioned about your father,

R: ... Yes sir,

I: That you notified them you had Kamiro, who made for you the final decision to go and they cut it, did you make that decision yourself or it’s your father who decided for you?

R: I my self decided because my dad for him he said us we are Baganda and us the Baganda don’t suffer those things, they are suffered by other tribes.

I: Why was he saying that those things are suffered by other tribes but not the Baganda?

R: Still for him he says among all the people in our clan there is no one whose Uvula has ever been tried to be cut. That he was only hearing it from other tribes that they are the ones who suffer from such.

I: Other tribes, what is it exactly?

R: Those other tribes besides Baganda, they are the one he says hears that there Uvulas get infected. And he told my son don’t go to those people lets first go the modern hospital but my wife for her she told that no at our place our Uvulas get infected lets go to Muzeeyi he is going to treat you she even told me you’re not the first, she told me there is a certain woman who came from that side of Entebe she came and her’s also was cut, I also got chance of seeing that woman when she had come to that thank that Muzeeyi. Because she brought for him sugar and a whole goat.

I: That Muzeeyi who cuts?

R: She actually brought for him a whole goat just to thank him, am so confident about this because I even saw it with my own eyes. Me when he finished cutting me he told me for you you’re my son the fact I gave him 5000sh...

I: ...Yet at first he had told you 60,000sh,...

R: Yes he had told me 60,000sh, but he later said you are my son whatever you will have in your pockets is what you will give me, and so I gave him 5000sh.

R: You didn’t go there with a hen?

R: No, I have never gone back there, I went back there only for introduction with my wife, that’s what I went back to do there.

I: Now, I heard you mentioning about your father saying that that thing only attacks those other people who are not Baganda, do you thing that thing is connected to witchcraft, that it can bewitched to someone?

R: I am not sure.

I: For him he just knows that it does not attack Baganda?

R: For it, it just attacks but i am not sure whether it’s connected to witchcraft.

I: So, from here to the traditional surgeon who cut your uvula, what’s the distance there, is it a mile or not?

R: Well it is.

I: Like how many miles?

R: Like one, starting from here?

I: Yes

R: It’s like one or one a half there.

I: Ok, so, during the operation, you mentioned about a razorblade,

R: Yes ,

I: That it was among the things which were used, but you also said it was trapped to pull it out. I want you to tell me, what else was used during that operation.

R: The fact, that thing...

I: ...you mentioned about hooking, what did he use to hook, did he hook with the same razorblade he cut with?

R: No,

I: Those are the ones I want you to mention about.

R: The techniques during then for sure I heard feared in person, he first told me to open my mouth and I opened, he broke the razorblade into two pieces...

I: ...half...

R: ...Yes half, but breaking it, there is something he placed it in, it was like leed...

I: ...a leed like this one...

R: ...well, am not so sure on what he placed that razorblade and he went on taking into my mouth, he told me to widely open my mouth, because I widely opened my mouth, then he hooked it out, it’s true he showed it to me, after removing it out there was a banana leaf he put it for me like this.

I: On the banana leaf...

R: Yes, on the banana leaf and I looked at it myself and he told me if possible take it to your home for them also to look at it, but I told him am taking it no where.

I: So you left it with him,

R: I left it with him.

I: Ok, now after being operated, you said that they gave you some Kisula salt to help you about that blood, what else were you given after you were operated?

R: Me because of my fear, for Muzeeyi he told go and salt and put it into warm water...

I: ...Our normal salt!

R: The normal salt I put in warm water and keep taking like two spoonful after every three hours.

I: Every after two hour to be taking two spoonful?

R: Yes, every after three hours I should be getting that warm water and keep taking two spoonful, but me because of my fear, the fact after him cutting it I went straight to the modern hospital.

I: Again to do what in the modern hospital yet you refused to go there to get treatment...

R: ...Me my fear was too much still I went there and explained to the nurse and I told him I have woods in the throat and he gave tabs which costed 4000sh and I tried and swallowed them because of the fear I had.

I: So now what about the salt, didn’t you use it a lot you mostly used the modern medicine the tabs?

R: Yes, because me I used the salt for only one day then I started using those tabs.

I: There is something you mentioned, you said after the four days, you were well and you could swallow very well, so I want you to assure me again since those four days up to now you are not feeling anything.

R: Well, I remained with some little pain, and I would eat matooke, cassava just like when having some wounds in there but I would eat food just like a normal person.

I: Now, you said after four days you started eating and feeling some little pain, I want you to tell me how many days did it take you to completely heal?

R: I for one it’s a week.

I: Week, within seven days you were ok...

R: ...i could not feel anything, I would everything could even take water with speed but unlike then before that thin was cut I couldn’t drink a cup water at once, I would drink like a child slowly.

I: You said you went the traditional Surgeon, when you reached there how long did it take before he cut it when you are there or he cut as soon as you arrived?

R: I found when he was still sleeping since I went to him very early in the morning at 6am, because it’s my father in law’s home so I couldn’t...

I: ...Eeh ! The Traditional surgeon is your father in law?

R: Because it where my wife if born, so I couldn’t just go there any how so I went to my brother in law’s place and for him he crossed the road and came to my brother in law’s place. He first looked into it and said my son you going to die this thing is too mature, when he came where I was it took like three minutes.

I: Okay, so how long did it take him to cut it?

R: Just seconds, according to the experience he is having it can’t even make an hour, it was at most 10 seconds, in case you open your mouth well, he told me to open my mouth well, at that time I gained the strength,

I: So, you said when you were leaving he gave you that water with Kisula in it,

R: No, it wasn’t water it was a paper...

I: ...it was in powder form after he had grinded it?

R: Yes, then he told me to try sucking...

I: ...When you were still at his place?

R: Yes, he gave some and told me when you reach home try to be sucking that kisula salt.

I: So when you reached home he had told you to take water with some salt...

R: ...some little salt after like three hours to take 2 to 3 spoonful.

I: So those 2 spoonful you are talking about of salty water, how much salt could you put in that water, could you put a full table spoon?

R: No, well I cooked a full cup of water in the saucepan but when I was going to take it I would warm it again because he told not to drink cold one, so i would warm it then swallow my 2 spoonful and then they cover it again very well, after three hour they warm it and I drink again.

I: The spoons you are talking about, do you mean the spoon which is used to serve sauce or the spoon which is used for tea?...

R: ...the one used for tea.

I: Okay, thank you so much, now you have been operated, I want you tell me how did you feel after you were operated?

R: When am still there at the traditional surgeon’s?

I: I want you to tell me, now you have come back from there we have reached home the operation is done, last time you were feeling so and coughing now let me know what happened.

R: At this moment or immediately when I got home?

I: On the day you came back and up until now.

R: When it was cut I went the modern hospital like I told you, I bought there some tabs and managed to swallow because of the too much fear and even my mother is the village VHT she told me son you have a very big mistake the throat is not played around with, we were supposed to go to a modern hospital to cut it but my son you have made a very bad mistake. Then I told her I will be healed, and actually they quarreled so much about that but God was me and I got healed so well I don’t have any complications.

I: So since then you got completely healed.

R: I got completely healed I don’t have any problems.

I: So the throat got completely well...

R: I don’t have any problem.

I: So after this operation, you don’t have any complication like maybe you got a scar you there which sometimes pains you...

R: ...no i don’t feel it, if it was there I would feel the damage whiles wallowing but I don’t have anything, I am fine.

I: So when he cut this Uvula, did he cot all of it or he only cut the half of it?

R: Now me who is not a traditional surgeon I can’t tell but in case he had cut it partly I think I would still be feeling it so it seems he cut it all?

……………………

1. ZIROBWE IDI_Client 02

I: So Mr. xxxxxn tell us about the situation you went through when your Uvula got infected, before it was cut, how was it, how was the situation?

R: That thing, me to start feeling the pain of that, it started in 2002, I go to the hospital, they inject me after one week the cough comes back, then I asked myself why, because I was injected thinking I was suffering from cough, and since my childhood I had never suffered from these things, and whenever i would do my work I remain steady, so I wondered what has happened to me , I went back and they again injected me and told the please the thing failed to get well, it became better for one week but its again back.

I: What do you mean the thing failed to get well?

R: The cough, it was paining me too much this coughing, and that coughing would bring me fever and I get to a bad condition, then I asked what is it, it even stopped me from working too much like I stopped doing heavy work, whenever I go to do heavy work I come back dying, then I asked myself what has happened to me, then one day I approached my grandparent, and I told him something is paining but I don’t understand it , then I asked what happened to you, I also told them I don’t know, then it was 2005 after I had married my wife, she was even pregnant there was a certain tree called omugavu, I married her when I even wonder about what I will eat if I don’t work so hard, will this cough stop me, and when we get children what am I going to do. Then I went back to my swamp to work to slash. It took me one week then my condition became bad again so I went back to the hospital, I asked myself am I not leaving the working now! Then they injected me the fourth time and I released me, but I did not do heavy work again then I started using money.

I: Using Money do u mean hiring work?

R: Workers, yes. Then I asked myself want am I going to do, am I suffering from HIV, but I said among the women I have been with no one has died of HIV, they are all looking healthy, I asked myself what is it, and I went back to the hospital and they took my blood sample I spent two hours waiting, then they checked three things, fever, TB and HIV when all the results came back I was found fine, then I asked myself what is it, still i wasn’t contented I spent like a week and I went to Koko.

I: What is in Koko?

R: Then I went there because I refused to accept the results from this hospital here, me I don’t so much believe in these clinics the nurses there are not well trained, so I went to Koko. When I reached to Koko, still they diagnosed three things from me, its me who ordered them because what I wanted is the final facts.

I: Still they diagnosed what and what?

R: Blood samples for HIV, TB and for fever, this am going to say I don’t know whether it will affect you...

I: No say everything.

R: I even paid there some little money and yet that is a government Hospital but I wanted them to do it for me very first by then I had no motorcycle at home. But still when the results came back I had nothing then I came back and I asked my neighbor here i have been diagnosed of three things but I don’t have any of them, what do you think am suffering from, I asked him is someone bewitching me but I again said do u have to just cough when you are bewitched and within me my heart refused, then I went back to my brother and my brother asked me to first go to Mr. xxe to examine may be the Uvula is the one infected .

I: Who Is Mr. xxx again?

R: The man is called xxxx from Kamwano the father in law of xxxxxx, he told me is he is going to examine me, he always examines during morning hours then he examined and told me I have it though I came not so prepared.

I: What thing did he say that you have?

R: The infected Uvula, that it’s the one that is making me cough and he told me it is very big and very soft and told me had I delayed for like five to six months it was going to burst because he told me I have delayed so much with it and he then told me to come back the following day with 8000sh, for sure i even did not have the 8000sh,so I went to my mother and she gave me the 8000sh. Then I woke so early morning, he told me to go very early in the morning when I have not eaten anything.

I: Musawo cccccc?

R: Yes, told me not to even take water, I got up so early morning and rode my bicycle up to ccccccc, so then I reached, the time it clocked 8am he had already removed it but the moment he placed it down there it was shaking but I couldn’t even have energy to talk and I even did not ask him why it was shaking, then he picked the thing he removed from me he too it and i saw him burying it...

I: ...Into the soil?

R: In the soil, he did not just throw it away, he dug a small hole put it in and covered it. Then when he came back I gave him the money then...

I: ...You left...

R: No, then he asked me,” do you have popcorns at your home? Then I told him I have the popcorns because me I was a maize farmer by then. I used too much to grow maize, minimum I would grow one acre but mostly I would start with three acres. So I came back and they fried for me the popcorns the ones they cook with ash then I ate, but on leaving he also gave me cotton then I put it into the ears.

I: What’s the purpose that cotton you put into the ears?

R: He told me that cotton prevents the air from blowing into the ears, but for me I slept with in it the ears, I did not remove it that that very day.

I: So when the air blows into the ears what does it do?

R: He told me there is where it’s going to affect me...

I: ...On the Uvula he has cut?

R: No, me he told me there is some way it will affect me, so since he told me that the air will affect me, I did not at any moment removed that cotton. So they had prepared my popcorns so every time I would be throwing into the mouth one by one of the corns...

I: ...Would you just swallow it or chew it?

R: No I would chew it. He had told me it cures the wound, me that’s how he told me. So me I did not swallow any tabs...

I: ...you did not swallow the modern tabs!

R: No, I did not try it at all.

I: Okay, now you told you went to Musawo dddddd and he cut it,

R: Yes

I: Who decided for you to go to him and how did you get to know about his existence and that he operates upon that uvula?

R: Mr. dcccccc is the one who told to ddddddd that he cuts Uvulas, go there and he examines you.

I: Oh! Okay, so now you have told me you went to Mr. cccccce he examined you and he saw that your Uvula had swollen and so he cut it, did he cut it using a panga or using a knife, I want you to tell me the things you saw him using to cut.

R: He brought a stick, it was like a reed and he broke a razorblade and he placed it in that reed, there is some way he made it as him which I can’t let you know at the moment because me I had just opened my mouth but here when he was finishing I saw he had broken the razorblade and there is away he cut, he told me to widely open my mouth, so when I opened he cut. Have you ever harvested a mango or a pawpaw?

I: yes

R: He knocks the first time and the second because he knocked like three times.

I: Knocking on the Uvula?

R: Yes, then he was done, he was handling something like a spoon inside the mouth, he trapped it, and by his trapping you also swallow the gas, so I swallowed the gas after then I saw him pulling it out and threw it there.

I: Now why was he trapping it?

R: Such that he handles not to fall inside...

I: ...he never wanted you to swallow it?

R: Yes

I: What do you think can happen to you in case you swallow it?

R: That one I don’t know, I did not ask him.

I: Now you mentioned about that thing of the razorblade,

R: Yes

I: Was the razorblade new or it was old?

R: It was a new one.

I: Did you see him unwrapping it?

R: Yes from there.

I: Is it you who took or you found it with him?

R: Himself

I: So, does Musawo xxxxxxxe work alone when operating or he has some helpers?

R: According to my knowledge he has some three or four people he trained, among those I can recall the names is his son, he also trained his musangi because they married from the same family he is called PKK, his son is called dxxxx and the other one stays up that side he has an Alur name which I can’t recall well, those are the only ones i know most the ones he trained here in dddxx village.

1. ZIROBWE IDI_Client 04

I: Please tell us about the condition called Akamiro that you experienced? How was it when u were suffering from uvulectomy?

R: It was all about severe coughing, I would cough and vomit, at times I would get itching in the neck and could not eat. I would also get a lot of neck and chest pain. It lasted for about two weeks. Even when I opened my mouth and the air enters I would feel bad. It was in the second week that I got a traditional surgeon. I went to him and he operated my uvula. When he finished he gave me salt water mixture to drink and some herbal medicine, and I went back home. When I came back I took food well without the pain I went through before the uvulectomy. The pain reduced after four days and I got my peace back. From that time, I have never felt any pain in the neck again or chest, and coughing stopped. I am fine

I: How would you cough?

R: I would cough endlessly and would spit nothing out but after the uvulectomy I got fine.

I: You said earlier that you would feel pain on the kneck when eating?

R: It was not pain but itching when coughing, when I cough I would vomit all the food, the uvula had taken long when it has swollen so it was the cause of the pain.

I: Whenever you coughed and vomited was blood in the vomits?

R: There was no blood in the vomits.

I: Would you feel pain in the chest whenever you vomited?

R: No I would not feel it

I: Now you have told me that the kneck would be itching you, so who decided that u had uvula and how did you know?

R: When I was coughing, those who saw me coughing and had undergone uvulectomy are the ones who told me. I had a neighbor who was an old woman and told me that I cough like someone suffering from uvula, then I told her that I did not know it, she requested me to open my mouth and check and the moment I opened my mouth she told me that my uvula had over grown. She directed me to the old Muluulu man where we went and he performed the uvulectomy

I: So you did not go to the government facility to get any medication?

R: I went to the clinic but did not get enough treatment, then I went to Bdddd in a government facility which is a HCII they gave me medication but it did not work, then I went to Kikyusa and took medicine for one week but still did not heal.

I: The medicine you were given, did they tell you what they were treating or not?

R: They were giving me medicine for cough.

I: Was the medicine a syrup or tablets?

R: It was tablets.

I: What did they tell you that those tablets heal?

R: They told me that it was for stopping the cough.

I: Now the old woman tell you that you have uvula, so how was it when you reached the surgeons place. Did he cut your uvula when you reached and at what time did you reach?

R: I went there very early in the morning.

I: What time was it?

R: It was around 7:00 am

I: So when you reached the surgeons place what happened?

R: When we reached there we found some people whom he worked on first. The after working on one of them he came and examined me then he confirmed that I was suffering from uvula, then he worked on those that had come before me and I was worked on last since I was the third one.

I: Ok you have told me that he worked on you, did you see the equipment that he used carry out the surgery?

R: He use a razor blade.

I: Was it full or?

R: It was not full, he breaks it into two

I: Did he use the same razor blade that he had used to operate the other clients?

R: No, he used my new razor blade on me, he uses a new razorblade for each individual.

I: So you mean you go with your own razor blade?

R: Yes, but if you do not have it he sends you to the shop to buy a new one.

I: So you are trying to say that the surgeon uses a new razor blade on each individual?

R: Yes, and he breaks it into pieces.

I: Can you explain how he uses that razor blade?

R: He ties it on a small metal

I: How does he make sure that you do not swallow the uvula when he cuts it?

R: He says that if he cuts the uvula and you swallow it, you automatically die, so he uses a string to tie it, the moment he cuts it he pulls it out. So he first hooks it before cutting it. Incase he cuts it without hooking it, it falls in the kneck.

I: When he told you that when he cuts the uvula and you swallow it you die, did you not get scared?

R: I did not get scared since I was already in pain and wanted it out and he was saving my life.

I: Does he work for free?

R: He works for money, at the time he was cheap, I paid UGX 3000/=.

I: You said you saw the razor blade and the metal, apart from those did you see any other equipment that he used?

R: No

I: Does this surgeon work alone or he has other individuals that help him?

R: He works with his sons, when you are scared they grab you such that you do not disturb him when cutting

I: Does this surgeon put on gloves or he just washes his hands before working on you?

R: When you find him doing his other works, he first washes his hands.

I: So after performing the uvulectomy did you bleed?

R: He tells you to wash your mouth with salt water and spit it out, then he tells you that that is all.

I: Is it cold or hot water?

R: It is cold water you find it when he has put it in the bottle.

I: So when you rinse with that salty water, is it helping you to stop bleeding?

R: I think so, I went up to home without bleeding I was just having some little pain and after I healed.

I: After the surgery for how long did you spend there when he is monitoring you?

R: It was like 30 minutes.

I: Did he put anything in your ears after the surgery?

R: Yes, he put some cotton wool.

I: It was for what use?

R: It was to prevent the wind from entering through the ears to the inner part of the kneck.

…………..

1. ZIROBWE IDI_Client 05

I: …………. So I kindly ask you to tell us about yourself.

R: My name is xxxxx, at first my child got a severe cough whereby he would cough as he vomits, then I took him to the health center where I was given a syrup and some tablets but he just continued having a persistent cough and he even got a strong fever. There some people who told me that what if he is suffering from uvula, then they told to take him to be checked whether he was suffering from uvula, then I took him for the check up and it was discovered that his uvula was infected. Then after I took him to local surgeon in rrrrrrrrr called Mr dddddd, I woke up very early in the morning and took him there, when we reached there the local surgeon checked him and he confirmed that the uvula had over grown, then he cleaned his equipment that he use they look like small sticks and he got a new razor blade and he broke it into 4 small pieces and he fixed it into the small stick he was cleaning and tied it.

Then he asked me how much I had brought for him then I told him I have only UGX 5000/= but he asked me to double it and I pay UGX 10,000/= then accepted and told him to perform the surgery. He then cleaned his equipment and he told me to hold my child firmly, then I held her in the position of breastfeeding and he opened her mouth then he used one stick to press the tongue down then after he brought the stick that had the razor blade and he fixed it in her mouth and cut the uvula and he removed it then he showed it to me. He told me to put the child upside down and she spit the blood out which had some clots then after he told me to breast feed my child immediately. She first refused to breast feed then later she breast fed well and after I paid him his money and we returned home. He also put cotton wool in his ears to prevent the throat from enlarging and being affected, he emphasized that we should not remove the cotton wool from the ears of the child, after that we came back home.

I: What is the use of that cotton wool in the ears?

R: The use of that cotton wool is that it prevents the throat from swelling and the swelling can lead to the death of the child, when the wind goes through the ears it affects the child.

I: You have talked about the razor blades used, were they new or old?

R: They are new razorblades, he opens it when you are seeing and breaks it into four pieces and one piece is used.

I: Does he use anything to hold the cut uvula and prevent it from falling inside the throat?

R: He use two sticks, one for holding the tongue and the other he fixes it into the mouth.

I: Does this tradition surgeon work alone or he is helped by other people?

R: He is always alone, the parent just holds the child firmly.

I: After performing the uvulectomy, does he give the child anything to drink?

R: No, he does not give anything, he just tells you to breast feed the baby and that acts as the medication.

I: After the uvulectomy what happened?

R: He just told me to overturn the baby to bring back the blood in the mouth and after he told me to breast feed her.

I: Was the blood she spit out a lot?

R: It was not much but had a few clots and some foam

I: How long did you spend at the traditional surgeon’s home?

R: We did not spend there a lot of time.

I: At what time did you take the child?

R; I took her there at 7:00 am since she had suffocated the whole night and could not breath well, so we left at 6:00 am.

I: So when you reached the surgeons place, did he perform the surgery there and then or?

R: When we reached he performed the surgery there and then, when he checked her he said the uvula had over grown.

I: What else happened after you had left the surgeons place?

R: Nothing much.

I: Apart from breastfeeding her what else did he tell you to give her?

R: Nothing, I just had to breast feed her. The fever stopped and the cough also stopped and she became fine. Actually, that night she had a peaceful night but before that he she would breast feed then cough and vomit. I used to give her the syrup from the health facility but they could not change much, before going to the local surgeon I first went to the health facility but it failed and people told me that the baby had uvula. One old woman on the village examined her and told me that she had uvula that is why I took her to the local surgeon. Actually she was examined by the wife to the area counselor who confirmed that her uvula was infected the she directed me to the local surgeon.

I: So when she examined her, what did she tell you?

R: She just confirmed that she was suffering from uvula and the uvula had over grown and directed me to take her for the surgery. But I knew the surgeon already since I had taken there my elder son for the surgery earlier.

I: You told me earlier that you went to the health facility first, what medication were you being given?

R: I was given syrup and tablets basically for the cough and fever since the uvula caused some fever.

I: Did she vomit the breast milk?

R: Yes, she used to vomit the breastmilk whenever she coughed and also got some fever.

I: What else happened to her, was she able to sleep well?

R: At night she would cough a lot and whenever she was breathing you would see depressions in his lower abdomen and you would see that she was in a lot of pain and she could not sleep. She would cry a lot as she throws herself down, when she takes the breast milk she would vomit the milk and you would see when she is in great pain as she cries, it would be worse at night. Basically at night she would cough a lot and she would breathe with difficulty and later vomit and the fever would attack her at night and the temperatures would go high. I would put on him a cloth with water and I would give her the tablets and syrup.

I: What happened to her body generally, did she lose some weight?

R: She did not lose too much weight and it did not last long since we addressed early.

I: How long did it take you to know that she had an infected uvula?

R: It took us only one week to find out but it was severe in the last 3 days then we discovered that she was suffering from uvula.

I: Did the traditional surgeon confirm it was uvula.

R: Yes he did so and performed the surgery and showed me what was going to kill my daughter

1. ZIROBWE IDI_Client 07

Tell us about the experience your most recent child went through while suffering from uvula

R: My last child who underwent uvulectomy started by suffering from strong cough that was not healing then he suffered from fever and high temperature, I took her to the health facility and she was admitted for some days when health worker claim that she had bacterial infection and was dehydrated. So they put her on a drip and after we came back home but still she was not fine. When she failed to heal I again to her to another health facility and they tested for malaria but it was negative and we brought her back home and we were advised to put a drip on her, by then she malnourished and her hair was all brownish and falling off the skull and the stomach was swollen. There was a lady who had earlier on examined my first child who had an infected uvula, so I decided to her to her for examination and when she examined her she confirmed that her uvula was infected then she directed to the traditional surgeon in Wabutungulu village. When we reached the traditional surgeons place he examined her and confirmed that the uvula had over grown and matured and about to burst, he told us that when it bursts the child automatically dies. When the uvula has over grown it develops purse within it. So he performed the uvulectomy that day and removed it. During the process of cutting the uvula it burst and poured on the tongue then he immediately asked for a cloth he used to clean the tongue and he told me to put the child upside down such that the child is prevented from swallowing the water from the uvula. Then after that I held her in normal position and he put cotton wool in her ears and gave us lemon and also told us to get ginger, that is all we used and the child got well.

I: You talked about the child having high heat, what do you mean?

R: I meant he would get high temperatures but when you reach the health facility and they test him, he would not be having any fever, but high temperature and he would not eat well since he would not be able to swallow food. The food would fail to down his throat actually that is when I took him to that lady for examination and after the uvulectomy she got fine and even the stomach that had swollen got back into shape and the hair got back to its original texture.

I: You mentioned that you went to a health facility first and was put on drip, what was the use of that drip?

R: He was dehydrated due to excessive vomiting and diarrhea with some green stool, whenever he would cough the uvula would scratch his throat and cause her to vomit and then for diarrhea the uvula has some dirty fluid it generates and cause her to have diarrhea so it was the cause of dehydration.

I: What cause the stomach to swell?

R: It was still this dirty fluid generated by the uvula because it is like pus when it has matured, so it drops inside hence the swelling of the stomach.

I: When you went to the health facility was the health worker able to identify that the child was suffering from uvula?

R: No, the health worker was not able to identify it, this is so because they just told me that the child had a bacterial infection and she is dehydrated and the child was admitted and put on drips for two days then she was discharged but still there was difference in the health of my child and by then the fever was high, she could not eat and was so weak and not able to walk properly. Then I took her to another health facility and they examined her but still found out nothing but he had high temperature with fever. So that is when I went to the old lady who had examined my first child who had the uvula to also examine this child too since she was experiencing the same signs like the first child of swollen stomach, brownish hair, strong cough diarrhea and vomiting, so when she examined her, she told me that the uvula had matured.

So when we went to the traditional surgeon to examine her, you know when examining they open the mouth and place a spoon to be able to see the uvula, so when he saw it he cut it, when cutting it they use a stick where they out the razor blade, he breaks the razor blade into two and put it on the stick and it forms a hook, he then puts the spoon in the mouth with the help of another person but also if you the mother can hold the child you do hold , if the mother cannot hold the child, she pays the money and they get somebody to hold the child then the surgeon makes the child to open her mouth and he places the spoon and use the hooked razor blade to cut it off.

I: What is the role of the woman that examined the child?

R: She is specialized in identifying the infected uvula, like now me I could not tell that the child had an infected uvula.

I: You said that during the surgery they used a razor blade, spoon and stick, was the razor blade ne?

R: Yes the razor blade was new, he broke it into 4 small pieces that can fit in the mouth of the child. Then he put that small piece on a stick so that he can be able to hook the uvula and cut it. Then he puts a spoon in the mouth of the child to open the mouth.

I: Who helped the surgeon to hold the child when performing the surgery on the child?

R: He got help from his colleague since I personally feared to hold my child, if you are not scared then you the mother hold the child, but for my case I feared seeing him when operating my child, so he called his wife to hold my child and I paid her.

I: You mentioned that the uvula burst in the process?

R: Yes it did burst since it had over grown and I had to face her down such that that fluid generated comprising of pus and blood is not swallowed. That is why held the child upside down and the surgeon got a cloth that he was using to clean the mouth of the child to avoid her from swallowing the content. Then after he put cotton wool in her ears to avoid further bleed since she is not supposed to inhale air through the ears to the throat.

I: How long does the cotton wool last in the ears?

R: It lasts like an hour usually it is removed when at home.

I: What is used to control blood flow?

R; He gave a lemon which I put in warm water and mixed it with ginger then give the child to drink, but the blood is not that much that come out. The ginger is crashed and dipped in warm water containing the lemon.

I: What amount of water does she take at the time?

R: It depends on the child but even two spoons are enough, I used to give her with a spoon, the use of the ginger and lemon is to speed the healing process of the wound. If it is an old child who can eat you do giver her popcorns with salt.

I: How long did it take this child to heal?

R: It took her only one day to feel fine, the next day she would eat and drink well.

I: What time did you go to the traditional surgeon?

R: I went at 4:00pm, you see each surgeon has different timing, now for the case where I took my first child he operates in the morning before the sun is over heat, when the sun is shining they do not operate since at the time the blood is flowing at a high speed, most of them operate in morning hours since by then the blood flow is low.

I: When you arrived at 4:00pm for how long did you wait to receive a service?

R: The moment we arrived and he examined her we did not wait much he broke his razor blade and operated her.

I: How did the surgeon prepare himself before working on the child, did he wash his hand and wear gloves?

R: He washed his hands with soap but did not put on gloves.

I: Is the surgery for free or you pay some money?

R: The one who operated my first child asked for a cock and some money, now for the one who operated my second child in Feb I paid him Uganda shilling 60,000/=.

I: You said when you reached home you gave this child warm water with ginger and lemon, what else did you give this child?

R: He could not eat that day so I just gave her that water, if the child is so young she cries the whole day due to pain.

I: Does the surgeon give anesthesia?

R: No, he does not do it, he operates direct.

I: So were you not scared?

R: Basing on the way the child was suffering and the health facilities had failed to find a solution and also the first child healed, so after the operation within one week the child is back to normal and healed so I was able to get the courage to hand over the child for the operation.

I: You said two of your children have gone through uvulectomy, do you have any of your relatives who has suffered from it before?

R: Actually its my two children who are the first ones in the family, I personally I had never heard of it.

I: What caused your children to suffer from Uvula?

R: I do not know the cause for sure, but the surgeon told me that the fact that the first child had and uvula all my children will suffer from it.

I: Do you think the uvula is associated to cultural issues?

R: I do not think so. But am also failing to understand it since in the health facility they do not understand uvula, even to some people it is hard to understand. I hear some people saying it is witch craft others say it is just a sickness and I personally I believe it is a sickness.

I: People who believe that the uvula infection is associated with witch craft, what could be their percentage?

R: They are 99% who associate it to witch craft. It is because this uvula is not understood in the health facility, like when I took my child to the facility they would only give me medication for cough but the cough cannot heal if the uvulectomy is not performed.

I: Did the child improve after the uvulectomy?

R: After the uvulectomy by day 2 she was very fine and ate food well and looked fine again.

I: How did the uvulectomy help this child? Like you mentioned earlier that she had a swollen stomach, brown hair and cough.

R: After the operation the cough stops that day, the fever stops, she stopped the diarrhea, the vomiting stopped as well and the swollen stomach after a week it goes back to normal. I had forgotten to tell you that when suffering from uvula the skin color changes of the child to yellowish so after the uvulectomy she returns to her original color and also feed well and finally the hair becomes black again.
