# Supplementary material for: Uvula infections and traditional uvulectomy: Beliefs and practices in Luwero district, central Uganda
Source: PLOS Glob Public Health. 2023 Jun 15;3(6):e0002078. doi: 10.1371/journal.pgph.0002078 (PMC10270344; doi:10.1371/journal.pgph.0002078)
Supplement: S4 Text — (DOCX) [file pgph.0002078.s004.docx]

**S4_Text_Excerpts from Transcripts of IDIs with Traditional Surgeons**

1. BOMBO_IDI_TS 01

I: What are your opinions on this condition called akamiro, what do you think about it?

R: It is bad, to sum it all it is dangerous, because you don’t get peace when you have it, it causes continuous severe coughing, one coughs nonstop without stopping, you have no peace until it is cut out and one gets better, at night one can’t sleep.

I: What other effects does it bring apart from over coughing and spending sleepless nights>

R: You lose weight and you may get malaria feverish and all may be there

I: so you might get fever?

R: yes

I: Why does a person lose weight?

R: A person may lose weight due to over coughing which may lead to discomfort, one doesn’t sleep, all the time you are worried, sincerely you lose weight.

I: But what do you think causes this Uvula infection/akamiro?

R: For sure I don’t really know what causes it, sometimes there are some babies who are born with it so I don’t know what causes it but it develops and the good thing it is operated once so it never appears again.

I: Now you have said that it grows/develops, am requesting you to explain more about this because we have gone to different places and different people have given us different views, so in your own opinion what is akamiro, where does it grow/originate from?

R: Okay, it is like this, it is short like this and everybody has it, but it can reach at a time and it becomes long and the more you grow it becomes longer so with this long size it reaches at time and it gets attached to the Goiter so this is when it begins disturbing after it has become very long. And when you stay with it and you don’t care about it, the color changes from red to black this is because it has over grown. Some people say that when it bursts you might die but I have never seen anyone with a raptured uvula.

I: So do the people you attend to come when it has out grown, what condition do they come in?

R: They come when they are seriously sick, over coughing, they don’t have peace, 2 days back there is one I cut, you will go there and ask him how he is feeling and he will tell you.

I: in what condition did he come in here?

R: He came, he came and was feeling bad and it can also be felt in the throat, you feel it tickling you in the throat, and when you are eating food there is a way it prevents food from going through the throat every time it is chocking you.

I: But what perceptions do people in your village have about it, what do they think about it because some people come and say please health worker it is this and that?

R: Some people come when they don’t know anything, some may come when they are over coughing, one can come and say, I have come so that you first check me because I feel my throat is itching and they say that if it is there you cut it out, so I first check and when I see that it is there that is when I cut it.

…….

I: On average how many people have you operated ever since you began doing this work?

R: Ever since I began

I: Yes

R: I don’t remember but they are so many

I: How many do you get per month on average?

R: It depends, because this is a rare infection where by you can spend a month without getting anybody but another month you get like 5 people another one like 2 people and it goes on like that.

I: Like 5 people a month

R: Yes like 5 people per month

I: According to you how is this number, is it high or low, what have you got to say about it?

R: This number for a month, I see it is small

I: Is there a period when you get so many people

R: Yes, once in a while I get like 8-10 people

I: What is usually the condition of the children by the time they bring them?

R: The children’s condition is similar to Asthma, they have difficulty in breathing and another thing with the child you can get to know very easily because when they breast fed the baby vomits everything even if it is food after eating they vomit

I: What about for adults do they vomit too?

R: No, they don’t vomit.

I: You talked about fever, pain and becoming black but now, you as a person who has done this job for a long time, what exactly causes this thing?

R: For sure I can’t tell you that it’s this which brings the infection because I don’t really know what causes it.

I: Why do you think that it is always necessary to cut it out from the throat?

R: I have never heard that when you use such and such medicine you can get better, no, but at the end of it all when they cut it that’s when you get peace/cured.

I: Have you ever received a person with it but you didn’t cut it out?

R: Yes I have ever but because he was scared so he went back home, It was due to fear.

I: What is the cause of this fear?

R: they fear and go back

I: mostly what makes them scared?

R: It’s the pain

I: Is there anything else that makes them scared?

R: No

I: Now how do you cut/operate the uvula from these people? What is the process like?

R: Operating, I get a piece of wood which helps to press the tongue so as to hold it down like this and then the Uvula will be clearly seen and the whole of it will lie on the wood, so when you cut it, it just comes out. I use a curved knife to cut it and while cutting it I press it on the wood and then I squeeze the knife to cut it down

I: Do you have to cut the whole of it or half way?

R: I cut it completely

I: or you can cut part of it

R: I don’t cut part of it, if you do so one will continue coughing

I: So how much do you charge them?

R: I charge them 10,000/=

I: Do they give you gifts?

R: No

I: How long have you been asking for only 10,000/=

R: I started from 5,000/= then I increased to 10,000/=

I: How long have increased the money?

R: Since last year

I: Isn’t there people who come and cry when they don’t have the 10,000/=

R: There those who come when they don’t have the money, you help them out. One may come with a child and the condition of the child is not good at all then you just have to be kind hearted because you are also a parent and you help them out.

I: You said that medical doctors in hospitals don’t understand it, but do you think these medical doctors have a way they can help people out.

R: No, this is because he cannot help you out when he doesn’t know anything about it, that is impossible he can’t help on what he doesn’t know.

I: A part from the other one you mentioned who calls you on phone.

R: Yes, he calls me I cut it and he provides them medical treatment and they go.

I: But why do you think people come to you to cut out the uvula/akamiro in the community? ..28:00…

R: It’s because they are told that it is not known in hospitals, they don’t know it and they are told there is one who can do it.

I: In hospitals they don’t know it?

R: they don’t know

I: But are there some people who come out and say I have moved a lot I went hospitals, I went to Mulago

R: They treat other things yet the actual ailment is different from what they treat.

I: So who directs them, how do these people get to know about you?

R: A person might be staying with in an area not here in Bombo or he might have lived here and shifted, so by chance she will tell you that she knows such and such a local surgeon who manages that kind of disease so try and take him there, then you will look for me and bring the patient to me.

I: Now if a person doesn’t treat this kamiro what will happen?

R: What can happen? Whatever would happen is neither upon me nor you but it is God who knows because when you treat it, you will be fine and still when you don’t treat it, you won’t be fine at all and its God who knows the results in the end. I can’t say that the person dies or not.

I: Okay, we have heard that when it bursts the person dies. But you have said you have not heard one who passed on

R: I had told you in the beginning, that I have never heard about anyone who died.

I: But have you heard about that too?

R: Yes, I have ever heard about it but I have never seen it myself. …………………………………..

I: According to you how long does it take to cut a person?

R: To me if you have not disturbed me with in a minute I may have finished you.

I: Even if it’s a baby?

R: Yes, but babies don’t disturb because if a mother holds it properly the way you have instructed her, they don’t disturb with in one minute am done.

I: anything apart from lignocaine that you would like to tell us?

R: That is what am still thinking about.

I: But when you are carrying out the operation, do you wear gloves?

R: Yes, I wear gloves and my knife is sterilized by boiling it every after ay surgery.

I: What else do you do to make sure that there are no infections on both sides?

R: Infections on the sick person

I: Yes, because the patient might infect you or you to infect him with any serious disease.

R: I use gloves. It is me who protects myself from getting infected because we are local surgeons in the community. You cannot know whether the patient has diseases like TB, you cannot know. We have to do it very fast. When a patient opens the mouth, you just cut it out very fast such that the patient goes away quickly without infecting you with his/her disease*.* if at all he has it.

I: Okay, unless you have something to say but we have come to the end.

1. BOMBO_IDI_TS 02

I: You said that it presents in a swollen manner, could there be something else swollen inside it?

R: Yes, if it is in that condition it causes tonsillitis side by side as it is in the middle then on both sides the tonsils appear like this that’s why someone feels hurt/sick.

I: What swells first, in your opinion?

R: What swells first? The uvula is already in place then I would say later on the tensile appears on both sides and they join. They come together.

I: then it comes out like this … okay, okay thank you. For the people who come, what do they have to say about it?

R: About the kamiro?

I: By the way what name do use to refer to it?

R: risi …

I: but then the community members who come what do they talk about it?

R: They talk good about me, when I attend to them and they get cured.

I: but what is their perception about that sickness?

R: There are some who come and tell me that; ‘‘I have moved to different health facilities but I don’t get cured, at first I took drugs but I don’t cured’’, so I attend to them. It is not that after attending to them that they immediately get cured, you can continue to cough but with time if you take drugs you can get cured but if you have that thing you keep on coughing. I swear it does not cure.

I: What do they tell you that they are suffering from, when they come, what complaint do they present, what complaint do they tell you?

R: I am feeling bad, I have difficulties in swallowing and sometimes I vomit when I eat, it chocks me, others don’t take long vomiting but they feel pain in this part of the throat.

I: Whom do they trust that has more expertise in attending to this sickness?

R: in this area we are three people I, yyy and Axxx, those are the ones trusted most because others are there but I don’t trust them, they try but… no they are fake they exist, they try sometimes they cut and cause damage/more harm, sometimes one is hurt and abandoned so he looks for another person to help treat him.

I: Do you often receive those that they have damaged?

R: Yes, but we had no intention of keeping these records, we didn’t think about registering the patients we attend to

I: So you usually receive them

R: yes

I: would they have cut it, what do they do exactly?

R: they damage it but do not remove it

I: what I wanted to ask, did they cut it

R: they cut I slightly, so when blood starts flowing, they stop before removing it. They cut it on top before getting to its roots/base.

I: Okay. There are two ways of operating; there is okay let me say it is of this size, then one cuts a little, then there is one who cuts deeply, so how do you cut it?

R: When you cut part of it better know that it disturbs, it is going to disturb that person, but we endeavor to cut all of it completely because when you cut part of it, it develops again but when you remove it, it doesn’t develop again.

I: Now, between the health care providers in the health facilities and you the local surgeons in the communities, who is more trusted by the people, where do they go more frequently to receive such kind of health care?

R: It depends on someone because there are those who don’t accept to come here well as others accept. Majority first visit health facilities, people go to health facilities first, if it fails then they look for another person elsewhere to provide the care.

R: Health worker, everyone comes with a different health complaint, there is one who comes complaining of not breathing well plus difficulty in swallowing, another one can come complaining of pain here I can’t do this, another one can come and is coughing, coughing all the time.

I: Okay, so each one presets with a different sign/symptom.

There are very many diseases but then why do you think this condition of Akamiro needs surgery? Why do you think the infected uvula/kamiro needs to be removed?

R: It is because he/she is not feeling well, they are not feeling well, if a person is not breathing well then he is not feeling well, what you need is to be attended to so that you are relieved back to the way God created you.

I: Okay, one is not feeling well, and would like to be saved. Okay, is there anything that can be done rather than operating or removing it?

R: That is back to you as health workers, you know what to do, maybe, could be, because you had not paid much attention to it but since you have started doing research about it could be there would be a way of healing without operating but time in memorial they have been operating. Those are health workers issues/concerns.

I: do you mean to say that whoever comes to you should be operated?

R: as local surgeons, we first observe, sometimes one may come with too much cough or saying ‘’this is how I feel’’ but us who know the cause of your coughing, when we check we find out if one has it or not and one without it we just leave her/him and tell them to go to the health facility for further examination. One could come without it.

I: but do they come knowing that they have it?

R: yes, but one without it goes back to the health facility for treatment.

I: How is the surgery performed, how do you operate or cut it?

R: I cut it. Some use a razor blade to cut it others use a certain machine/tool, me too I use my own tool. One time I went to a health worker and inquired so she explained to me that do this and this, I was with health worker Laker Mustafa. Log ago we never used to fear but when HIV/AIDs came in we stopped operating thinking we might kill people so we went to the health worker who explained to us so well about what to do so we resumed and saved people’s lives properly.

I: What did they teach you?

R: how to boil and keep it safely

I: okay, may I have a look at it?

R: you will see it

I: You as an individual how do you avoid contracting any disease from this patient who has come, we have seen there many diseases, and again blood would be flowing, blood has issues.

R: I put on my gloves, now that we use masks I use my mask too, only that. I don’t touch any blood or what, I don’t touch it.

I: why don’t you touch blood?

R: because it is not too much blood flowing that you need to wash someone, no and for a young child it can vomit so if it happens then it is the mother who will clean up and an adult cannot vomit so I can’t touch in blood and this person is not covered with blood.

I: If blood start flowing in your capacity what do you do?

R: I burry it, the person spits out and I burry it and this piece of fresh which I cut off I burry it properly

I: and that is to the time you were provided with tins, so why don’t you hand over that piece of fresh to them?

R: To take it at home?

I: yes

R: if one wants to take it I do give it to them so they take it but most of them don’t take it

I: You know I have asked because some of these people who go for circumcision say that, ‘‘I want to take my thing’’. That they are going to make lipstick out of it

R: lipstick?

I: well it is just rumors we don’t know whether it is true, that’s why I inquired about this one too, so you burry them.

What challenges do you face during surgery?

R: I have not had any challenges but there are some people who disturb, they disturb, but I work with people who hold them and I cut it, but ever since I began working I have not got any problem.

I: you said that you have worked for 25 years, were you wearing gloves in the past too?

R: we would do things without wearing gloves so when HIV/AIDs came I went there and the health worker taught me, right now I put on my gloves

I: Which health worker taught you?

R: It was a health worker in Gatinda but he passed on, he was in the facility there.

I: Do you sometimes become suspicious of some people and you don’t attend to them?

R: Yes, but with that sickness if one has it you can’t know whether you are suffering from HIV/AIDs or not. One time a man came and looked very thin so one could think he is infected with HIV/AIDs, so I said, you man go to the hospital, ‘mum I have moved, I have moved a lot’, but as you are looking bad, then he said ‘mum I am not infected’ I said you can’t appear like that you must be infected, ‘mum please attend to me if I am to die it is okay but I have moved to many health facilities and I have not succeeded’’. So I said I am going to charge you a lot of money because I will not use all my tools again, ‘mum please attend to me’. So I charged him 5,000/= and in the past it was a lot of money, 5,000/= was too much, so I operated him and he left. After a few months he returned but I had forgotten him so he said that, ‘mum do you remember me?’ I said no I don’t remember you, he said ‘you look at me closely’, I said I don’t remember you, he said that one time I came here to be helped and you told me that you are infected with HIV/AIDs, I asked, are you the one? He said, ‘I am the one mum’. He had come with his son, he said that the sickness I had which was disturbing me has affected him too. I operated him, my God I wonder where that man is, but whenever he sees me, ‘mum you are my God on earth, what, what’

I: it means this condition had weakened him

R: on his appearance you could immediately think it is HIV/AIDS, it really affects someone’s health so much and leads to loss of weight so that’s when you can know that this sickness kills, it is so painful too and deadly.

………………………

1. BOMBO_IDI_TS 03

……

I: What are your opinions about this condition ‘akamiro’? or what do you talk about it this condition ‘akamiro’?

R: What I know about this disease it brings a persistent severe cough and sometimes it causes pain in the ribs, and this disease we call Asthma since there is difficult in breathing from the lungs and this always brings difficulty in breathing and also vomiting when one has eaten food, sometimes it also raptures when one over coughs, one can cough and it raptures.

I: what raptures?

R: the akamiro itself

I: But what is exactly this akamiro condition/Uvula infection?

R: Mum sincerely that thing I also found it there, for sure I don’t know because by the time I was born it was already there but in treating it I used to realize a difference and according to the people I treated also they recovered and got better compared to the condition they were in before. There are some who come and before the treatment they couldn’t speak for a long time without coughing. Sometimes when they have lost too much weight, others come from hospitals but when the results are saying they have no TB, no what but when they have been directed, surely when they can’t even walk but now after the treatment they have recovered and still living.

I: When they have failed to recover from the hospital?

R: Yes, when they have tested but failed to recover from the sickness but when the person is losing weight and energy.

…….I: But what do you think causes the akamiro condition?

R: With that for sure I don’t know, because some infants have it but also old people grew up with it, and when they become adults it starts to disturb them, so with that I can’t tell you that it is caused by this, its inborn because I think everybody has it.

I: Everybody has it when it is infected or not?

R: It’s like this, everybody has it but it depends some are small and they can easily disturb because every time you breathe it moves, it’s light, some are big, this one rarely affects people though it could be infected too.

I: What could have happened to it so that it is in a critical condition that you decide it should be cut out?

R: Madam that thing develops and whenever you cough it develops, it changes the color and it becomes red so it ends up rupturing passing out some fluids. But when it ruptures most of them may not live any more, rapturing depends on the way the someone is coughing?

I: Why do you think the fluids that come out of it ca lead to death?

R: I really don’t know why and I think it’s you the medical people to help us in that.

I: Have you ever heard or seen anybody who had an Uvula infection that when it ruptured that person died?

R: They are many, they die.

I: At what stage does it normally rapture?

R: It has no stage it raptures but it depends on the pressure used in coughing, that is when it raptures but there is someone I operated just like that one I mentioned who was almost losing his life because he was in a critical condition by the time I removed it, it had turned from the red color the blood which was coming out of it had changed the color to black.

I: The blood was black?

R: Yes, and inside it there are small particles which look like the eggs of a fish

I: like this, like small stones?

R: yes

I: What do people in this community that you see talk about the akamiro?

R: In my area, I hope most of the people know about it and people come from different villages for treatment

I: But what do they talk about it, because when you get to the health worker, you begin to explain the condition you are in so when they get here what do they complain about what has happened?

R: Sir, I have come here I have persistent cough, sometimes they are feeling pain, it is painful, poor eating habits because whenever they eat the food comes back

I: But to them, do they come when they know what is really affecting them?

R: Yes, they come well knowing what is paining according to people who direct them so they come when they know that they have an Uvula infection and when we remove it, they leave after seeing what was killing them, they go with it, they don’t leave it behind.

I: Do you have people who first diagnose them before sending them to you?

R: No

I: What perceptions do people have about this sickness which you are managing? What do they think about the sickness?

R: Madam, people are there relaxed because they well know that this disease exists, okay there are some who don’t know but one may fall sick like that one they got from the hospital, there is a person who directed him that so and so does this, so it is the people who direct them to come here. But for them they don’t have any...

I: Which people are trusted most in managing this condition?

R: come again

…

I: Who is most trusted in managing this condition?

R: Madam we are all experienced and trusted, I think there is no one they don’t trust

I: According to the local surgeons in this community?

R: Yes

I: Okay, what about you the local surgeons and the ones at the health facility?

R: Doctors at the hospital have not reached the level of treating it because they claim that they don’t know the disease at all.

I: Why do you confidently say that they are not aware of it at all?

R: Because we have a hospital here in the barracks which is at the level of a municipal and many health facilities but I haven’t heard of any treating it.

I: Okay. Normally these people you treat here when they have akamiro what advice do you normally give them? you call it RIS

R: RIS

I: what advice do you commonly give them?

R: The advice I give them, after operating and treating that thing, at that moment I get salt (ekisula) and mix it with water, then the patient gaggles it in the mouth and spits the water. The treatment we give, we tell them to eat food because when they eat food that salt passes on the wound. If one has taken food within one day the person may be fine people can’t even know that one was operated upon but those who don’t want to swallow saliva keep on spitting every time and by the time he wants to swallow the saliva the throat may be dry.

1. ZIROBWE_IDI_TS 01

……….

I: What is their perception on this condition of akamiro?

R: For us the local people we have nothing to think about, we know the sickness exists, where possible seek for treatment for the patient, if you are not able refer to the another person who can provide treatment, this sickness is like hernia I cannot treat hernia it’s the ones who know it that can do.

I: In our opinion who do most people trust in managing this condition?

R: I have nothing to tell you, I may be working from this end and I get a person coming this end, then so and so may be the other side and he also receives one, if they know that this one can help us.

I: If you compare the health facilities and managing it locally?

R: In my capacity I have nothing to say because even in the health facilities you could be aware of it and you carry on operations, sometimes.., you see most people around here have so many sicknesses, there are sickness managed in health facilities whereby it is impossible if you seek treatment locally, then there are sickness where you visit health facilities and they tell please we are failing try the local health workers in villages so such sicknesses are there.

I: You mentioned earlier someone who came from Mulago, if you could compare do most people first visit the health facilities and on failure to heal come to you or?

R: Yes, after failing, they usually say I have taken medicine, done what, I have bought drugs for fever and provided to the child but it has failed.

I: What advice do you usually provide to people who present with akamiro?

R: I, actually for us we don’t have a school that I will teach that do this or that, when they bring a patient I just provide treatment, I provide medicine and then go, and make sure it doesn’t reappear.

I: What drugs to you usually give them?

R: A lot, we have our local medicine and it is what we use, you give it t them and the drink it.

I: After you have attended to this person what else do they do?

R: After working on them, they give me token of appreciation

I: how much is that token of appreciation?

R: it is up to them, it has no limit

I: you may charge little

R: we negotiate

I: so you negotiate

R: yes

I: but then I can think of too little money, as a patient can think of very little charges, don’t you have a limit where you say that I don’t go below this?

R: no, I don’t have

I: why don’t you have specific charges?

R: because sometimes they just give us chicken, and you also take it, your aim is to heal a person.

I: Then how about things to be done after the operation, for instance if you worked on a baby, you give medicine to the mother?

R: I provide droplets to the baby

I: what else should the mother do for this child?

R: should breastfeed

I: how does breastfeeding help?

R: should breastfeed right away, immediately should begin to breastfeed, if at all it was breastfeeding?
