# Supplementary material for: Uvula infections and traditional uvulectomy: Beliefs and practices in Luwero district, central Uganda
Source: PLOS Glob Public Health. 2023 Jun 15;3(6):e0002078. doi: 10.1371/journal.pgph.0002078 (PMC10270344; doi:10.1371/journal.pgph.0002078)
Supplement: Attachment — Submitted filename: Response to reviewers.docx [file pgph.0002078.s005.docx]

21^st^ April 2023

Steve Zimmerman, PhD
PLOS Staff Editor,

**RESPONSE TO REVIEWERS PGPH-D-23-00282**

Dear Dr, Zimmerman. Thank you for your positive review of our manuscript titled “Uvula Infections and Traditional Uvulectomy: Beliefs and Practices in Luwero District, Central Uganda
PLOS Global Public Health” We have addressed both the journal and reviewers’ comments and are pleased to re-submit the revision. Please see specific points below. Thank you for your consideration.

**Journal Requirements:**

a. Please review your reference list to ensure that it is complete and correct. If you have cited papers that have been retracted, please include the rationale for doing so in the manuscript text, or remove these references and replace them with relevant current references. Any changes to the reference list should be mentioned in the rebuttal letter that accompanies your revised manuscript. If you need to cite a retracted article, indicate the article’s retracted status in the References list and also include a citation and full reference for the retraction notice.

***Response:*** We have made updates for full citations of the references, removed double entries and dited the errors that were existing with formatting and typos. These are indicated below.

This was edited to a complete reference.

Hörmann K, Verse T. Surgery for Sleep Disordered Breathing. Second Edition ed. Berlin Heidelberg: Springer; 2005

This has been edited to one complete reference and its duplicate has been removed.

Prual A, Gamatie Y, Djakounda M, Huguet D. Traditional uvulectomy in Niger: A public health problem? Social Science & Medicine. 1994;39(8):1077-82. doi: <https://doi.org/10.1016/0277-9536(94)90379-4>.

This has been edited with typo removed.

Kunii O, Tanaka Y, Lewes A, Wakai S. Uvulectomy and Other Traditional Healing Practices: Traditional Healers' Perceptions and Practices in a Congolese Refugee Camp in Tanzania. Tropical Medicine and Health. 2006;34(4):159-66. doi: <https://doi.org/10.2149/tmh.34.159>.

We have added a DOI to this reference

Owibingire SS, Kamya ER, Sohal KS. Beliefs about traditional uvulectomy and teething: awareness and perception among adults in Tanzanian rural setting. Annals of International Medical and Dental Research. 2018;4:25-30. doi: 10.21276/aimdr.2018.4.2.DE6.

We have edited the following as well that had formatting issues.

Alebachew BW, Minuye BB, Yeshambel AA. The burden of traditional neonatal uvulectomy among admissions to neonatal intensive care units, North Central Ethiopia, 2019: A triangulated crossectional study. PLoS One. 2020;15(7):e0234866. Epub 20200709. doi: 10.1371/journal.pone.0234866.

Lange S, Mfaume D. The folk illness kimeo and "traditional" uvulectomy: an ethnomedical study of care seeking for children with cough and weakness in Dar es Salaam. J Ethnobiol Ethnomed. 2022;18(1):35. Epub 20220429. doi: 10.1186/s13002-022-00533-9. PubMed PMID: 35488269.

Owibingire SS, Kamya ER, Sohal KS. Beliefs about traditional uvulectomy and teething: awareness and perception among adults in Tanzanian rural setting. Annals of International Medical and Dental Research. 2018;4:25-30

Ndu IK, Duke-Onyeabo CP, Onu NN, Nnamani OG, Iheji CC. Traditional Uvulectomy: A common and potentially life-threatening practice in a developing country. Medical Science and Discovery. 2022;9(8):450-3. doi: <https://doi.org/10.36472/msd.v9i8.779>.

**Reviewer #1:**

a. This article draft looks at practices related to traditional uvulectomy, a phenomenon that appears to be relatively common in several African countries, but that is under-researched. The authors write that to the best of their knowledge, this is the first study to look at this form of folk surgery in Uganda. The article is therefore an important contribution to this field of study. The article does not relate to a conceptual framework or theory. The main strength of the article draft is the triangulation, which gives us the perceptions of both the folk surgeons, community members, uvulectomy clients, as well as community health workers. The findings are very interesting, and they are discussed in relation to existing literature on traditional uvulectomy. I highly recommend this article draft for publication, but some issues need to be addressed before publication:

***Response:*** Thank you for this encouraging comment. We have addressed the issues as recommended below, and hope the article qualifies for publication.

b. Ethics: In the transcripts of the interviews, the full personal names of all the participants are given. Did they give their consent to this? If yes, have the researchers considered any negative consequences, particularly for the lay surgeons? Even if the practice is not restricted by law today, that may happen later (not the least after the publication of this article). I recommend that all the personal names are removed from the transcripts if they are made publicly available.

***Response:*** Thank you for noting this anomaly. This was a gross oversight. For the supplementary file, we have stripped out any identifying information and kept excerpts from mainly transcripts with sections where the quotations were picked from. We attach one supplementary file containing excerpts from interviews, well labelled. This will protect the participants confidentiality as promised, especially the traditional surgeons and community health workers.

c. Community health workers: Please provide some more background information about this cadre in the Ugandan health system since the term “community health worker” is used for both salaried and volunteer health workers in different contexts. What kind of training/education do they have? Are they based at local level health facilities or do they just operate from their homes?

***Response:*** We have added the roles of CHWs and provided a reference to the guidelines for the reader, in case they need detailed information beyond what the manuscript offers. The CHWs in Uganda are known as Village health Team members (VHTs). They are incorporated into the formal health system as the lowest health delivery structure, also known as health centre 1. They comprise of volunteers that are selected by communities from within themselves. CHWs are offered on-job training and refresher sessions by Ministry of health and implementing partners in health. Their roles include supporting the primary healthcare function, offering health information, and linking community members to the health services. Lines 120 to 127

**Methods:**

d. Page 5, line 101-15: The authors state that the two sub-counties are “predominately occupied by Baganda” (…) “with Bombo Town Council including a large Nubian community”. Did the authors ask the participants about their ethnic affiliation? It would be useful to have some reflections regarding the degree to which the uvulectomy practices are linked to certain ethnic groups or not.

***Response:*** Thank you for this comment. Unfortunately, these data on ethnicity were only collected in the quantitative survey. We are preparing a manuscript for that to submit for publication as well. The survey data indicate that the Nubian community had the highest prevalence. However, we cannot include that data in this qualitative manuscript.

e. Page 5, line 106-109. The authors describe all the interviews as “in-depth”, but do not say anything about the length of the interviews. Information about the length should be included in the revised manuscript (no need to state the exact length of each interview and FDG, but a time range should be given).

***Response:*** Thank you for this addition. We have added a statement from line 166 to line 169 Interviews with clients and surgeons were mainly to supplement the survey, with brief topic guides. These interviews with clients lasted an average of 30 minutes, while those with VHTs were shorter lasting an average of 25 minutes. FGDs lasted an average of 1 hour and 17 minutes.

f. From the attached transcripts, we see that of the eight transcribed interviews with community health workers, four of them are only 3-4 pages long. Similarly, of the IDIs with clients, three of the eight interviews are 5-6 pages long. Normally, to qualify as an in-depth interview, the interview should last approximately an hour (a minimum of 30 minutes), and if this had been the case the transcript would be much longer in my experience.

***Response:*** Thank you for this guidance about in-depth interviews. We note that the Interview guides were short focusing on providing explanations to the survey tool, for this mixed methods study. We have added a statement about the average time for interviews and FGDs in line 166 to 169 as indicted in response above to provide clarity to the reader. We have also corrected them to interviews instead of In-depth interviews, given the guidance provided.

g. I also note that the FDGs in Zirobwe are considerably longer than those in Bombo, and in Bombo, the participants generally give very brief responses. This makes me wonder whether this is linked to the fact that different research assistants worked in the two locations. I also wonder whether the transcripts are indeed verbatim (word by word) or whether the research assistants perhaps summarised the interviews in the original language (Baganda) during the translation process to English? Did any of the authors listen to the recordings to check the quality of the translated transcriptions? Perhaps some reflections on this can be added to the limitations section.

***Response:*** Thank you. We note the short responses in Bombo. This could be because of the more peri urban nature, and people may not provide the same amount of time as the rural Zirobwe. However, yes, SPSK (corresponding author) reviewed transcripts and listened to a sample of adui files (1 from each research assistant). The interviews were conducted by the same team across Bombo and Zirobwe. The excepts from interview are missing some text. The shorted interview transcript was 8 pages in length. We have added the potential for minimal loss of text to the limitation to the paper. From line 502 to 505. There is a risk of loss of information during the transcription as translations are done from Luganda to English, given that the transcribers are not native English speakers. A quality control was done by SPSK, although this may not entirely eliminate the risk.

h. Page 5, line 112-113: “The participants were carefully selected to include…”. Exactly how were the participants recruited?

***Response:*** We have added more clarity about participants selection to the methods section, from line 141 to 149. “Persons who underwent traditional uvulectomy or the caregivers were identified from the household survey and asked if they wanted to share some more information about their experience. The CHWs are known in the villages, and were selected based on availability as long as they were active in their roles. Traditional surgeons were also known persons well identified by the community members. They were approached to voluntarily participate after explaining the study purpose. FGD members from the communities were selected with the support of the CHWs who supported mobilisation. They should have been residents of the community for at least 12 months.”

i. Page 6, line 120-134: This section describes the issues that were covered in the topic guides but does not say anything about why the researchers decided to ask these specific questions. For example, from the transcripts we see that participants were asked about the possible connection between TB and akamiro. Was this based on a pre-study, information from Stellah Kayongo (“who alerted the university about the existence of traditional uvulectomy”, page 21), or was it based on reading of the existing literature on traditional uvulectomy?

***Response:*** The questions in the guides were aimed at supplementing the survey in answering study objectives. The tools focused on beliefs and perceptions about Uvula and Uvulectomy (which this paper presents), outcomes and sequelae as well supplement the magnitude of Uvula infections. The questions on Tuberculosis (TB) only came in as probes by the Research assistant, after identifying persistent cough lasting some time, one of the symptoms that is communicated to the public as a possible sign of TB. They were not part of the main tool.

j. Language: Overall, the manuscript is very well written and structured. However, there are a few errors that need to be corrected (please note that there may be others, since this reviewer is not a native English speaker).
   Page 2, line 36: “using audio recorder key informants interviews” – should be “recorded”?
   Page 2, line 41: “the sized chicken heart” – should be “the size of a chicken heart”?
   Page 5, line 117: “by a team four well…” – should be “team of four…”?
   Page 11, line 236: “My child has ever suffered from it” – should be “has suffered from it”?
   Page 13, line 295: “there is away he cut” . should be “a way”?

***Response:*** We apologize for some of these lazy mistakes. We have re-read the manuscript and made edits to the English grammar and removed spelling mistakes, and made other edits, as seen in the tracked changes version.

k. I wish the authors all the best for these minor revisions and look forward to seeing the published version.

***Response:*** Thank you for the best wishes. We have made the adjustments that you suggested. Your review has greatly improved the manuscript indeed.

Thank You,

**Simon Peter Sebina Kibira**

Corresponding Author
